# The Therapeutic Potential of Essential Oils in Managing Inflammatory Skin Conditions: A Scoping Review

**DOI:** 10.3390/ph17050571

**Published:** 2024-04-29

**Authors:** Anouk E. W. K. Dontje, Catharina C. M. Schuiling-Veninga, Florence P. A. M. van Hunsel, Corine Ekhart, Fatih Demirci, Herman J. Woerdenbag

**Affiliations:** 1 Department of Pharmaceutical Technology and Biopharmacy, Groningen Research Institute of Pharmacy (GRIP), University of Groningen, Antonius Deusinglaan 1, 9713 AV Groningen, The Netherlands; anoukdontje@gmail.com; 2 Department of Pharmacotherapy, -Epidemiology and -Economics, Groningen Research Institute of Pharmacy (GRIP), University of Groningen, Antonius Deusinglaan 1, 9713 AV Groningen, The Netherlands; c.c.m.schuiling-veninga@rug.nl (C.C.M.S.-V.); f.vanhunsel@lareb.nl (F.P.A.M.v.H.); 3 Netherlands Pharmacovigilance Centre Lareb, Goudsbloemvalei 7, 5237 MH ‘s-Hertogenbosch, The Netherlands; c.ekhart@lareb.nl; 4 Department of Pharmacognosy, Faculty of Pharmacy, Anadolu University, 26470 Eskisehir, Türkiye; demircif@gmail.com

**Keywords:** acne vulgaris, case reports, clinical studies, dermatitis, eczema, efficacy, essential oils (EOs), psoriasis, rosacea, quality, tea tree oil (TTO)

## Abstract

Conventional therapy is commonly used for the treatment of inflammatory skin conditions, but undesirable effects, such as erythema, dryness, skin thinning, and resistance to treatment, may cause poor patient compliance. Therefore, patients may seek complementary treatment with herbal plant products including essential oils (EOs). This scoping review aims to generate a broad overview of the EOs used to treat inflammatory skin conditions, namely, acne vulgaris, dermatitis and eczema, psoriasis, and rosacea, in a clinical setting. The quality, efficacy, and safety of various EOs, as well as the way in which they are prepared, are reviewed, and the potential, as well as the limitations, of EOs for the treatment of inflammatory skin conditions are discussed. Twenty-nine eligible studies (case studies, uncontrolled clinical studies, and randomized clinical studies) on the applications of EOs for inflammatory skin conditions were retrieved from scientific electronic databases (PubMed, Embase, Scopus, and the Cochrane Library). As an initial result, tea tree (*Melaleuca alternifolia*) oil emerged as the most studied EO. The clinical studies with tea tree oil gel for acne treatment showed an efficacy with fewer adverse reactions compared to conventional treatments. The uncontrolled studies indicated the potential efficacy of ajwain (*Trachyspermum ammi*) oil, eucalyptus (*Eucalyptus globulus*) oil, and cedarwood (*Cedrus libani*) oil in the treatment of acne, but further research is required to reach conclusive evidence. The placebo-controlled studies revealed the positive effects of kānuka (*Kunzea ericoides*) oil and frankincense (*Boswellia* spp.) oil in the treatment of psoriasis and eczema. The quality verification of the EO products was inconsistent, with some studies lacking analyses and transparency. The quality limitations of some studies included a small sample size, a short duration, and the absence of a control group. This present review underscores the need for extended, well-designed clinical studies to further assess the efficacy and safety of EOs for treating inflammatory skin conditions with products of assured quality and to further elucidate the mechanisms of action involved.

## 1. Introduction

As an introduction to this scoping review, we first (Section 1.1) provide general information about aromatherapy (the definition and history) and essential oils (EOs) (the definition, production methods, chemistry, and dermatological applications) followed by the aim of this study. Second (Section 1.2), we present the background information (the clinical picture, pathophysiology, and treatment options) of the inflammatory skin conditions this article focuses on: acne vulgaris, dermatitis and eczema, psoriasis, and rosacea.

### 1.1. Essential Oils and Aim of this Scoping Review

Among consumers and healthcare providers, there is an increasing interest in using essential oils (EOs) (also known as volatile oils, ethereal oils, or aetherolea) from plants for medicinal purposes [1,2]. The application of EOs for therapeutic purposes is known as (clinical) aromatherapy [3]. Aromatherapy comprises the inhalation, topical application, and enteral intake of EOs, involving, respectively, the olfactory system, the skin, and the digestive tract as the route through which an EO can exert its physiological and pharmacological effects [4]. Aromatherapy is used for the treatment and prevention of diseases and to obtain a positive effect on mood, emotions, and psychological well-being. The French perfumer chemist Rene-Maurice Gattefosse coined the term ‘aromatherapy’ in 1928. While working in his laboratory, he burned his hands and found that lavender EO helped the healing of the wounds with little scarring. Further significant steps in the development of aromatherapy were set by the physician Jean Valnet, who, in the 1960s, expanded aromatherapy to include wider medical applications. More recently and still active, Robert Tisserand is an important scientist and ambassador for the safe and therapeutic use of aromatherapy and EOs [3,5,6,7,8].

EOs are mostly volatile lipophilic liquids, which are, according to international definitions (including pharmacopoeias), obtained from aromatic plant material (leaves, flowers, fruits, and roots) mostly by hydrodistillation (or steam distillation), as well as by expression (in the case of *Citrus* spp.). The word ‘essential’ refers to the ‘essence’ of a plant (volatiles, odor, and flavor) captured in the EO. The extracts of aromatic plants that are obtained using organic solvents (known as concretes or absolutes) or using supercritical carbon dioxide are not considered to be true EOs, although they may be marketed as such. Their composition is different from EOs, both qualitatively and quantitatively, and, in addition to volatile (EO) constituents, they will contain a number of non-volatile compounds as well. Thus, their pharmacological and safety profiles may differ from EOs [3,5,6,9,10].

The constituents of EOs originate from three distinct biosynthetic pathways: the mevalonate pathway and the methyl–erythritol pathway leading to terpenes and terpenoids, respectively; and the shikimic acid pathway resulting in volatile phenylpropanes. The number of individual substances analyzed in EOs is generally large (>300), resulting in a great variation in their composition. EOs may contain one or a few main constituents. EOs typically consist of monoterpenes (C10), volatile sesquiterpenes (C15), and phenylpropane derivatives with a molecular mass up to around 300 Dalton. Based on their chemical structure, they are aliphatic or cyclic hydrocarbons, alcohols, aldehydes, ketones, acids, esters, or phenols [3,5,9,10]. EO-producing plants are concentrated in a distinct number of plant families. EOs exhibit various biological activities, including antibacterial, anti-inflammatory, and antifungal [3,5,6,9,10]. These specific properties make several EOs beneficial for the treatment of inflammatory skin conditions.

EOs are rarely applied undiluted onto the skin as such a direct application can cause severe irritation. Commonly, in practice, they are diluted with a carrier oil (oleum, vegetable oil, fixed oil, or fat), a cream base, or a gel. Usually, concentrations of 2–5% are used in practice, but strongly irritating EOs are applied in maximal dilutions of 0.5% [2,7,8,11]. This reduces the rate of evaporation of the EO and may increase the rate and depth of its penetration into the skin. Transdermal absorption may occur as well, resulting in systemic effects including possible acute and chronical adverse reactions. A hydrophilic base, such as a gel, may enhance the skin penetration compared to a lipophilic base. Additionally, a base may also enhance the therapeutic benefits based on its skin healing, moisturizing, soothing, and nourishing properties [8].

This present scoping review aims to generate a broad overview of EOs used in a clinical setting to treat inflammatory skin conditions such as acne vulgaris, dermatitis and eczema, psoriasis, and rosacea, in order to define their clinical potential. The focus is on the frame of the clinical studies, the safety aspects, and the quality of the EOs and the administered preparations thereof. First, in the next section, the relevant background information on the inflammatory skin conditions is provided. 

### 1.2. Inflammatory Skin Conditions

Acne vulgaris is a common and persistent inflammatory skin condition of the hair follicle and sebaceous glands [12] mainly occurring in the age group of 12 to 24 years due to hormonal changes during puberty. The pathophysiological factors include an increased sebum production, the hyperkeratinization of the hair follicles, inflammatory factors, and bacterial overgrowth, primarily involving *Cutibacterium acnes* (formerly known as *Propionibacterium acnes*) [13,14]. Acne vulgaris is characterized by the persistent or recurring presence of comedones, pustules, and red papules on the skin of the face, upper limbs, and neck [12]. The treatment options for mild-to-moderate acne vulgaris include topical treatment with retinoids, benzoyl peroxide, and/or topical antibiotics. Often, erythema and dryness of the skin are experienced as undesirable effects, reducing adherence to these therapies [15].

Eczema is a form of dermatitis characterized by symptoms such as skin redness, swelling, scaling, oozing or weeping, and dryness. The most prevalent type is atopic eczema, a chronic inflammatory skin condition causing patients to experience a decrease in their quality of life. Exacerbations may cause emotional stress among patients. Various factors are involved in the development of the condition including a compromised skin barrier, the enhanced activity of immune cells in the skin, the composition of the skin microflora, and external factors, such as low environmental humidity [16]. Another form of dermatitis, seborrheic dermatitis, manifests in areas of the body with a high concentration of sebaceous glands, especially on the scalp. Consequently, the condition is prominently visible and requires treatment to improve the patient’s quality of life [17,18]. Topical corticosteroids are frequently used in the management of dermatitis and eczema and are proven to be effective. However, extended use of these drugs can lead to undesirable effects, including skin thinning, stretch marks (striae), dilated blood vessels (telangiectasias), and reduced skin pigmentation [19]. This may cause poor patient compliance, leading to treatment failure [20]. 

Psoriasis is a persistent immune-mediated inflammatory skin condition [21]. The most prevalent form of psoriasis is (chronic) plaque psoriasis. It is characterized by erythematous scaly patches or plaques, commonly located on the extensor surfaces of the knees and elbows, as well as the scalp, but can also affect the intertriginous areas, such as the palms, soles, and nails [21,22]. Lesions may appear at sites where the skin experiences trauma or pressure with the concomitant risk of bacterial infection [23]. Most psoriasis patients experience a negative impact on their quality of life, with many reporting significant psychosocial distress [23]. The current treatment options for mild psoriasis include topical treatment with corticosteroids, vitamin D analogs, and keratolytics, as well as phototherapy [23]. These strategies aim to alleviate symptoms, enhance the quality of life, and curb the condition progression. However, adverse drug reactions, resistance to treatment, long-term safety concerns, and the high costs associated with certain therapies may restrict their utilization and negatively influence patient compliance [24]. 

Rosacea is a chronic inflammatory skin condition characterized by one or more primary features, including flushing (or transient facial redness), persistent central facial redness, inflammatory papules or pustules, and telangiectasia [14,25]. It mainly affects the centrofacial regions, including cheeks, chin, nose and forehead, and eyes. Rosacea may adversely affect the quality of life, including social and psychological well-being [26]. While a complete understanding of rosacea’s pathogenesis remains elusive, there is evidence suggesting a complex etiology, involving genetic, immunological, and neurovascular factors, and various triggers, including pathogens, ultraviolet radiation, and dietary factors [27,28]. Due to the presence of these multiple contributing factors, the management of rosacea can be challenging [28]. 

Patients suffering from one of the above-mentioned inflammatory skin conditions may face disappointing results from the conventional treatment options and/or experience adverse drug reactions leading to premature termination of the therapy. If so, patients may be willing to try an alternative or complementary therapy, for instance, in the form of an EO, in the hope of better clinical outcomes and fewer adverse drug reactions [3,7,8,9,29,30,31,32]. In this context, it is worth mentioning that a rising global trend has been found in the use of complementary medicines among psoriatic patients, especially in semi-urban areas where such therapies are favored due to their accessibility and affordability, as well as their presumed effectiveness and safety [33].

## 2. Results

### 2.1. Scoping Review

**Figure 1:**
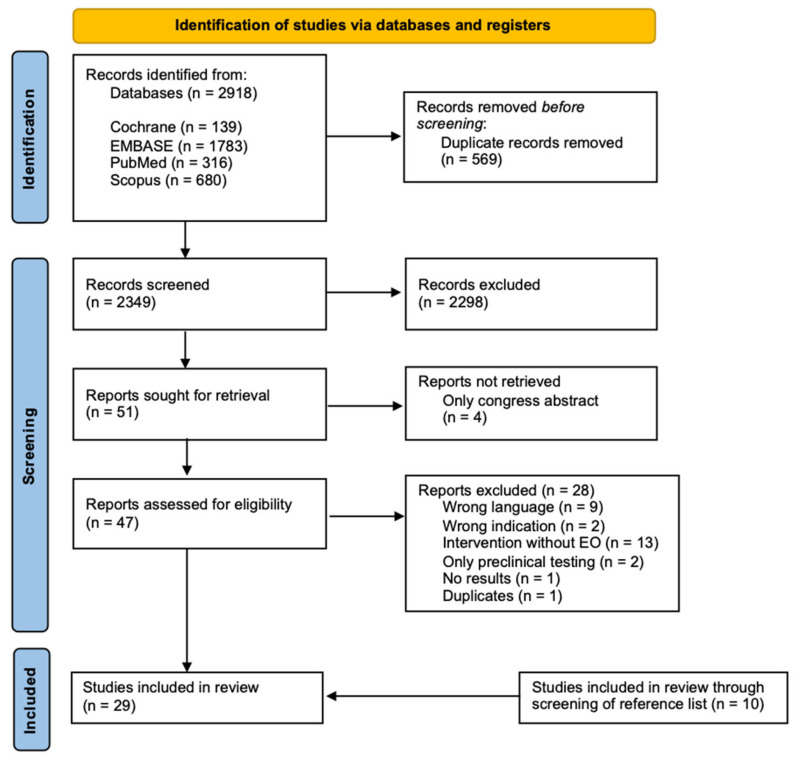
PRISMA flow diagram of included articles in this scoping review [34].

A total of 2918 studies (covering all publication dates) were initially identified. After removing duplicates (n = 569), 2349 titles and abstracts were screened for the inclusion criteria. As a result, the full texts of 51 studies were sought for retrieval. Unfortunately, the full texts of four articles were unavailable and, therefore, excluded. A total of 47 studies were assessed for eligibility, and 28 were excluded for the reasons provided in Section 4.2. Based on the reference lists of the studies assessed for eligibility, ten additional studies were included. Consequently, a total of 29 studies were included in this scoping review. Sixteen of the eligible studies focus on acne, four on dermatitis, five on psoriasis, and one on rosacea. Additionally, three eligible clinical studies without specifics resulted from the search strategy. The PRISMA flow diagram of the study selection process is presented in Figure 1 [34]. The composition of the EO-containing preparations, as described in the respective references, is shown in Table S1 (Supplementary Materials).

### 2.2. Acne

Table 1 gives an overview of the characteristics, main results, and limitations of the included studies focusing on acne. Table 2 gives an overview of the characteristics of the products used in the included studies focusing on acne to assess their quality. The studies cover a range of interventions, with tea tree (*Melaleuca alternifolia*) oil (TTO) as the primary EO used in the various formulations. The key findings include the superior effectiveness of benzoyl peroxide over TTO in reducing inflamed lesions, the positive outcomes of TTO in treating mild-to-moderate acne, the efficacy of cinnamon bark (*Cinnamomum verum*) oil in reducing acne lesions, the potential of cedarwood (*Cedrus libani*) oil in improving acne, and the antimicrobial effects of tea tree and lavender (*Lavandula* spp.) oils. The other studies evaluate the effectiveness of different formulations such as clindamycin–tretinoin, *Aloe vera* with African basil (*Ocimum gratissimum*) oil, Unani herbomineral cream, and the EO from copaiba (*Copaifera officinalis*). Overall, these studies indicate promising results for various EO-containing treatments in managing acne, with generally minimal ADRs (adverse drug reactions) reported in the studies. The studies are presented and discussed in order from the highest to lowest power of clinical evidence.

A double-blind, placebo-controlled study conducted by Enshaieh et al. aimed to assess the effectiveness of TTO in treating mild-to-moderate acne vulgaris in young patients. The study included 60 patients, divided into two groups receiving either a 5% TTO gel or a placebo gel. The TTO group experienced a significant reduction in acne lesions, total lesion count (TLC), and acne severity index (ASI) compared to the placebo group, and the TTO gel was found to be significantly more effective. Few ADRs were reported in both groups including minimal pruritus (five subjects; 8.3%) and a little burning sensation (three subjects; 5%). In the study, the origin of TTO was declared to be Melaleuca alternifolia (from Cinere Company, without any further specifications), while the physical and chemical properties of the commercial products containing TTO were stated to be regulated by an international standard [35]. Therefore, no additional analyses were performed by the authors on the purchased intervention and control products [36]. 

Kwon et al. conducted an 8-week, double-blind, randomized, controlled split-face trial with 34 participants, comparing the clinical efficacy, safety, and histopathological changes between 5% of *Lactobacillus*-fermented *Chamaecyparis obtusa* (LFCO) in cream and 5% of TTO in cream for treating acne vulgaris. The LFCO reduced inflammatory acne counts significantly after one week, while the TTO did so after four weeks. The LFCO also reduced non-inflammatory acne lesions and significantly decreased sebum levels. Additionally, the LFCO was more effective than the TTO in reducing the lesion counts. A significant difference between both groups in inflammatory lesion counts and acne grading was observed after two weeks and for non-inflammatory lesion counts after four weeks. In the TTO group, mild dryness (four subjects; 12.5%), mild erythema (six subjects; 18.8%), and desquamation (six subjects; 18.8%) were reported as ADRs. In the LFCO group, mild erythema (two subjects; 6.3%) and dryness (two subjects; 6.3%) were reported. The origin of the TTO was declared to be *Melaleuca alternifolia* (obtained by the steam distillation of leaves and terminal branches), while the LFCO originated from the needles (leaves) of *Chamaecyparis obtusa*, a type of cypress frequently found in Korea and Japan which is known as hinoki oil. Both intervention and control products were purchased from commercial sources. However, the production process was described and additional analyses for quality control were conducted such as ultra-performance liquid chromatography (UPLC) and high-resolution mass spectrometry analysis [37].

Da Silva et al. investigated the use of 1% of EOs from copaiba (*Copaifera langsdorffii*) in natrozol gel for the treatment of mild acne. The study, a double-blind, placebo-controlled clinical trial, involved ten volunteers with grade one (mild) acne lesions. The results showed a significant decrease in the extent of the area affected by acne in both the intervention and placebo groups, but the effect was more significant in the intervention-treated group. ADRs were not monitored or documented in this study. The intervention product was obtained from copaiba oil resin through steam distillation and used at a concentration of 1% in natrozol gel. Analyses, like high-resolution gas chromatography, were conducted on the EO for quality control, whereas the control was purchased without additional analyses [38].

The study conducted by Bassett et al. aimed to compare the efficacy and skin tolerance of a 5% TTO gel with a 5% benzoyl peroxide lotion for the treatment of mild (grade one) and moderate (grade two) acne. This prospective, single-blind study involved 124 patients divided into two groups receiving either TTO gel or benzoyl peroxide lotion. Both treatments effectively reduced the number of inflamed lesions. However, benzoyl peroxide was significantly more effective than TTO after one, two, and three months of treatment. TTO was better tolerated on the facial skin than benzoyl peroxide, and minimal ADRs were reported including skin dryness, pruritus, stinging, burning, and redness. The intervention and control products were purchased, and no additional analyses were conducted [39].

Ghovvati et al. investigated the safety and efficacy of a topical cinnamon bark EO (*Cinnamomum verum*) formulation for mild-to-moderate acne. An open-labelled, assessor-blind, uncontrolled clinical trial was conducted including 20 patients receiving 0.5% cinnamon gel. This gel reduced acne lesions, especially inflammatory ones, and lowered sebum content. Minor yet tolerable ADRs were reported including mild burning (13 subjects; 65%) and facial redness (11 subjects; 55%). Subsequently, the topical gel (an ethanolic carbomer gel) was prepared, and additional analyses for quality control, including the chemical quantification of the formulation, were conducted [40]. 

Talebi et al. evaluated the safety and efficacy of a topical ajwain (*Trachyspermum ammi*) EO formulation for treating facial acne through an open-label, uncontrolled, phase 2 clinical trial including twenty volunteers. The study’s outcomes included a therapeutic efficacy assessment, a clinical evaluation, an assessment of patient satisfaction, the measurement of biophysical skin parameters, and the monitoring of ADRs. The ajwain gel showed a statistically significant reduction in acne lesions, especially non-inflammatory ones, as well as improvements in the skin characteristics. Local ADRs were monitored according to the authors, but none were reported in the article. The ajwain gel was extracted and prepared by the researchers and additional analyses for quality control were conducted by gas chromatography coupled with mass spectrometry (GC-MS) [41].

Malhi et al. aimed to evaluate the efficacy, tolerability, and acceptability of 200 mg/g TTO gel and a 7 mg/g TTO face wash for treating mild-to-moderate acne. A dual-center, open-label, phase II pilot study with fourteen participants was conducted. The TTO gel significantly reduced lesion counts and acne severity. Additionally, the adherence of the participants was high. They expressed satisfaction with the reduction in lesion counts. The TTO products were well tolerated with only mild local tolerability events including dryness, scaling, and peeling. The Australian TTO in both purchased products met the specifications of the British Pharmacopoeia monograph for TTO and both the ISO 4730:2004 and Australian 2782–2009 standards for ‘Oil of *Melaleuca*, terpinen-4-ol type’. Furthermore, a visual determination of each product’s minimum inhibitory concentrations (MIC) against *Cutibacterium acnes* after 48 h of anaerobic incubation was included in the study [42].

In the study of Hassan et al., the effect of eucalyptus (*Eucalyptus* spp.) oil in a fatty cream basis on the treatment of acne was investigated involving five patients. The study reported good results and improvements in the volunteers’ acne conditions after the second week of treatment. Any ADRs were not evaluated. The constituents needed to prepare the eucalyptus oil cream, and the constituents were purchased and subsequently mixed without additional analyses [43]. 

Najafi-Taher et al. conducted a triple-blind, randomized clinical trial with 100 mild-to-moderate acne vulgaris patients. The aim was to evaluate the efficacy and safety of a TTO nano-emulsion (6%) containing adapalene (a topical retinoid) gel (0.1%) compared to a marketed adapalene gel. The TTO nano-emulsion in combination with adapalene gel was more effective than the marketed adapalene gel. The ADRs were mild and similar in both groups with dryness being the most common ADR. The TTO was purchased and subsequently manufactured into a nano-emulsion. The additional characterization of the products, and in vitro, ex vivo, and in vivo analyses were conducted [44,45].

Mazzarello et al. evaluated the efficacy of a cream containing propolis, TTO, and aloe vera (PTAC) for mild-to-moderate acne compared to a 3% erythromycin cream and a placebo. There were no specifications reported about the cream base. A double-blind, comparative study with 60 patients divided into three groups was conducted. The PTAC was more effective in reducing acne lesions, erythema, and sebum compared to the erythromycin cream. No ADRs were reported. The intervention and control products were purchased without conducting additional analyses [46]. 

Mazzarello et al. compared the anti-acne effectiveness of clindamycin–tretinoin with a cream containing *Myrtus communis* and *Origanum vulgare* EOs and tretinoin (MOTC). The study design was a comparative, randomized, single-center, single-blind study involving 60 patients. Both treatments improved the clinical parameters, including the number of comedones, papules, pustules, TLC, and ASI, with no significant differences between them. However, the MOTC had a better efficacy in reducing the papular erythema. No ADRs were documented. Both the intervention and control products were acquired from commercial sources without undergoing supplementary analyses [47].

Parveen et al. evaluated the effectiveness of an Unani herbomineral cream (UHC) for treating acne vulgaris in a controlled, randomized, single-blind clinical trial. The control and test groups each included 30 patients. UHC is a complex product containing several EOs in addition to other herbal products. The UHC demonstrated effectiveness in reducing acne lesions over 60 days compared to the control cream. The ADRs were not monitored or reported. The UHC was self-made by the researchers. According to the authors, the quality was assured on the basis of the descriptions given in the botanical sources and the classical Unani literature. No further analyses were reported [48]. 

Kim and Shin examined the effect of treating acne with a basic, weekly acne treatment (not specified) and a dermal application of a mixture of 3% TTO, 2% lavender (*Lavandula* spp., no further specification) oil, and jojoba (fixed) oil. The study was designed with a non-equivalent control group design with 60 participants divided into control and intervention groups. A significant reduction in the total number of *Cutibacterium acnes*, the sebum excretion rate, and non-inflammatory skin lesions was observed in the intervention group but not in the control group. In contrast, the number of acne lesions was significantly reduced in both groups. However, the reduction in the number of human skin pathogens did not significantly differ between both groups. The ADRs were not monitored or reported. The EOs used were permitted by the Korean Food & Drug Administration and assessed by the standards of the Korea Laboratory Accreditation Scheme. No additional analyses were reported [49]. 

Orafidiya et al. aimed to determine the effective concentration and base for a topical African basil (ocimum) (*Ocimum gratissimum*) oil formulation for managing acne vulgaris, compared to benzoyl peroxide. The clinical study with 126 subjects assessed the reduction in lesion counts and skin tolerance. The preparations containing ocimum oil in alcohol and a cetomacrogol base were the most effective in reducing the lesion counts. The reported ADRs in the intervention group were minimal and limited to a burning sensation, whereas no ADRs were reported in the placebo group. The intervention and control products were obtained from commercial sources without performing additional analyses [50]. 

Another study conducted by Orafidiya et al. aimed to investigate the synergistic effect of *Aloe vera* gel on the anti-acne properties of ocimum oil and compared them with clindamycin. The study included 84 participants. Ocimum oil lotion with aloe gel exhibited the most significant effects in reducing lesion counts. The higher the aloe gel content, the greater the product activity. The aloe gel-based preparations were the most active in resolving acne lesions. As many as 96% of the patients reported a mild stinging sensation on the skin and a mild skin irritation. Again, the intervention and control products were purchased without conducting additional analyses [51]. 

Hassoun et al. reported a case study discussing a 35-year-old woman with acne vulgaris. Cedarwood (*Juniperus virginiana*) oil was added to tretinoin cream, and the acne case was improved with no ADRs being reported. The commercial cedarwood oil was purchased and self-added to the tretinoin cream by the patient [52].

**Table 1:** Characteristics, main results, and limitations of the included studies focusing on acne. Details about the composition of the preparations are given in Table S1 (Supplementary Materials).

Author(s) (Publication Year; Country)	Study Design (n)	Intervention (n)	Control (n)	Outcome Measure(s)	Study Duration (Evaluation Period)	Main Results	Limitations
Enshaieh et al. (2007; Iran) [36]	Randomized, double-blind, and placebo-controlled study (60)	5% TTO ^1^ in carbomer gel (30)	Carbomer gel (30)	—Total lesion count —ASI ^2^ —Number of comedones, papules, and pustules —Monitoring ADRs ^3^	45 days (baseline and every 15-day period)	—After treatment, a significant reduction in the mean TLC ^4^, ASI ^2^, and the numbers of comedones, pustules, and papules in the intervention group was observed but not in the control group (significant difference) —5 patients reported minimal pruritus, 3 patients reported a little burning sensation, and 1 patient reported minimal scaling (divided over the intervention and placebo groups)	—Short duration of study and follow-up time —Small sample size
Kwon et al. (2014; South Korea) [37]	Prospective double-blind, randomized, and controlled split-face trial (34)	5% *Lactobacillus*-fermented *Chamaecyparis obtusa* (LFCO) in cream (34)	5% TTO ^1^ in cream (34)	—Acne grade —(Non-)Inflammatory lesion count —Histopathology and immunohistochemistry of lesions —Patients’ subjective assessment —Average size of sebaceous glands —Sebum secretion —Monitoring ADRs ^3^	8 weeks (baseline; 1, 2, 4, and 8 weeks)	—A significant difference between both groups in inflammatory lesion counts and acne grading was observed after 2 weeks and for non-inflammatory lesion counts after 4 weeks —A significant reduction in the overall size of the sebaceous gland was observed on the lactobacillus side when compared to the baseline, but not on the tea tree oil side —In the tea tree oil group, 4 patients reported mild dryness and 6 reported mild erythema and desquamation —In the lactobacillus group, 2 patients reported mild dryness and 2 reported dryness	—Risk of cross-contamination —Lack of comparison to a control or placebo —Small sample size and all were Korean
Da Silva et al. (2012; Brazil) [38]	Double-blind and placebo-controlled clinical trial (10)	1% Copaiba essential oil in natrozol gel (10)	Natrozol gel (10)	—Photographs to determine the total area occupied by acne	21 days (baseline and weekly)	—After 21 days, a highly significant decrease in the extent of the area affected by acne was observed in both the placebo and intervention groups; however, the effect in the intervention group was greater —The results observed in the placebo group could not be explained, and aggravations occurred in most of the participants —The decrease in the surface area affected with acne in the intervention group can mostly be explained by the model	—Short duration of study and follow-up time —Small sample size —Only one outcome —No safety assessment —No demographics reported
Bassett et al. (1990; Australia) [39]	Prospective and single-blind comparative study (124)	5% TTO ^1^ in gel (61)	5% benzoyl peroxide in lotion (63)	—Severity of acne (counting technique) —(Non-)Inflammatory lesion count —Oiliness, erythema, scaling, pruritus, and dryness —Monitoring ADRs ^3^	3 months (baseline and three times with monthly intervals)	—At the baseline, a greater facial erythema assessment was observed in the tea tree oil group compared to the benzoyl peroxide group —At 1, 2, and 3 months, benzoyl peroxide reduced a greater number of inflamed lesions compared to the tea tree oil group; however, the reduction in both groups was significant —There was not a significant reduction in non-inflamed lesions observed in both groups —79% of the benzoyl peroxide group reported ADRs ^3^, whereas 44% of the tea tree oil group reported ADRs ^3^ (a significant difference)	—Lack of control or placebo group
Ghovvati et al. (2019; Iran) [40]	Open-label, assessor-blind, and uncontrolled clinical trial (20)	0.5% cinnamon in gel (20)	NA	—(Non-)Inflammatory lesion count —Size and quantity of follicular fluorescence spots —Skin biophysical parameters —Monitoring ADRs ^3^	45 days (baseline; 4 and 8 weeks)	—ADRs to 1% cinnamon in gel were observed in 6 subjects; therefore, the 0.5% cinnamon in gel was used in this trial —After 8 weeks, significant reductions in the non-inflammatory and inflammatory lesion counts, sebum content, and erythema index were observed compared to the baseline —Non-significant changes were observed for the skin hydration and melanin index —13 patients reported mild burning and 11 patients reported facial redness after using the cinnamon gel	—Lack of comparison to a control, placebo, or standard therapy —Short duration of study and follow-up time —Small sample size —No demographics reported
Talebi et al. (2020; Iran) [41]	Open-label, assessor-blind, and uncontrolled clinical trial (20)	1% (*w*/*w*) ajwain in gel (20)	NA	—Digital and fluorescence photography —Biophysical skin profile measurement (stratum corneum hydration, sebum content, TEWL ^5^, erythema index, melanin, and pH value) —TLC ^4^ —VAS ^6^ —Monitoring ADRs ^3^	8 weeks (baseline; 4 and 8 weeks)	—After 8 weeks, a significant reduction in the total lesion count, non-inflammatory lesion count, and a mean decrease in the size and quantity of red spots was observed —After 8 weeks, the decreases in sebum and pH values were significant —No ADRs ^3^ were reported	—Lack of comparison to a control, placebo or standard therapy —Small sample size
Malhi et al. (2017; Australia) [42]	Uncontrolled, dual-center, and open-label phase II pilot study (14)	200 mg/g of TTO ^1^ gel and 7 mg/g of tea tree face wash (14)	NA	—IGA ^7^ score —TLC ^4^ —Skin oiliness —Frequency of ADRs ^3^ —Mean local tolerability score	12 weeks (baseline; 4, 8, and 12 weeks)	—After 4, 8, and 12 weeks, a significant reduction in the TLC ^4^, IGA ^7^ score, and facial oiliness score was observed —Adherence was high, and no serious adverse events were reported	—Lack of comparison to a control, placebo, or standard therapy —Short duration of study and follow-up time —Small sample size
Hassan et al. (2013; Saudi Arabia) [43]	Clinical trial (5)	Eucalyptus oil in cream (5)	NA	—Photographs —Outcomes not defined	10 days (monitored routinely)	—All patients showed good results after the second week of treatment	—Lack of comparison to a control, placebo, or standard therapy —Short duration of study and follow-up time —Small sample size —No specific outcomes reported —No safety assessment
Najafi-Taher et al. (2022; Iran) [44]	Triple-blind and randomized clinical trial (100)	TTO ^1^ nano-emulsion in adapalene gel (53)	Adapalene gel (47)	—Number of (non-)inflammatory lesions —TLC ^4^ —ASI ^2^ —Monitoring ADRs ^3^	12 weeks (baseline; 4, 8, and 12 weeks)	—Number of (non-)inflammatory lesions, TLC ^4^, and ASI ^2^ showed a greater improvement in the intervention group compared to the positive control —Only mild ADRs ^3^ were reported in both groups, with dryness being the most common adverse effect	—Lack of comparison to a control or placebo
Mazzarello et al. (2018; Italy) [46]	Single-center, randomized, and double-blind comparative study (60)	—Group A: 20% propolis, 3% TTO ^1^, and 10% *Aloe vera* (PTAC) in cream (20)	—Group B: erythromycin cream (20; positive control) —Group C: vehicle (20; placebo)	—Cutaneous status —Macro-photography —Quantity of sebum —Skin surface pH —Cutaneous erythema index —Number of comedones, papules, and pustules —TLC ^4^ —ASI ^2^	30 days (baseline; 15 and 30 days)	—All the clinical and instrumental values studied were statistically different from the placebo group —The sebometry, pH, and erythema index values were not significantly different statistically during the treatment in the three study groups —The intervention of group A was more effective in reducing ASI ^2^ and TLC ^4^ compared to the intervention of group B —The interventions of both groups A and B showed a significant reduction in the number of (non-)inflammatory lesions	—Short duration of study and follow-up time —No safety assessment
Mazzarello et al. (2020; Italy) [47]	Comparative randomized, single-center, and single-blind study (60)	3.74% *Myrtus communis* L. essential oil, 0.1% *Origanum vulgare* essential oil, and 0.025% tretinoin (MOTC) in cream (30)	1% clindamycin and 0.025% tretinoin (CTG) (30; cream)	—Amount of skin sebum, pH, erythema index, and TEWL ^5^ —Macro-photography —Number of comedones, papules, and pustules —TLC ^4^ —ASI ^2^	30 days (baseline; 15 and 30 days)	—All the instrumental values studied were statistically different between the two groups; however, no sebometry and pH changes were observed in both groups —The MOTC intervention was better than the CTG control in reducing the popular erythema index —In the CTG control group, erythema of the inter-papular healthy skin significantly increases statistically —Both products improved clinical parameters, and no statistically significant differences were detected between the groups	—Lack of comparison to a placebo —Short duration of study and follow-up time —No safety assessment
Parveen et al. (2009; India) [48]	Controlled, randomized, and single-blind clinical trial (30)	Unani herbomineral in cream (20)	Vehicle of cream (10)	—IGA ^7^	60 days (baseline; 15, 30, 45, and 60 days)	—The IGA ^7^ means show a very significant difference between the baseline and day 15, day 30, day 40, and day 60 after treatment, whereas very significant differences in the placebo group were observed between the baseline and days 45 and 60 —No significant difference in the IGA ^7^ was observed between the baseline and days 15 and 30 in the placebo group	—Lack of comparison to a standard therapy —No safety assessment —Low number of outcomes
Kim and Shin (2013; South Korea) [49]	Non-equivalent control group, pretest–post-test design (54)	A basic, weekly acne treatment and a dermal application of a mixture of 3% TTO ^1^ and 2% lavender oil in jojoba oil as the carrier (27)	Basic, weekly acne treatment (27)	—Acne-related characteristics —Number of P. acnes —Number of human skin pathogens —Number of acne lesions —Sebum secretion rates	4 weeks (baseline and weekly)	—A significant reduction in the total number of P. acnes, the sebum excretion rate, and non-inflammatory skin lesions was observed in the intervention group but not in the control group —The number of human skin pathogens showed no significant difference in both groups —The number of acne lesions was significantly reduced in both groups	—Short duration of study and follow-up time —Small sample size —No safety assessment
Orafidiya et al. (2002; Nigeria) [50]	Preliminary clinical tests (126; seven subjects per product)	*Ocimum gratissimum* leaf essential oil preparations (1% *v*/*v* polysorbate 80, cetomacrogol blend, and petrolatum base, or 50% *v*/*v* alcohol) (112)	—Reference: 10% benzoyl peroxide in lotion (7) —Placebo: 40% arachis oil in 1% *v*/*v* polysorbate 80 (7)	—Number of comedones, papules, and pustules (acne lesion count) —Number of days taken to achieve a 50% reduction in lesion count —Skin sensitivity —Patient acceptability —Monitoring ADRs ^3^	4 weeks (baseline and weekly)	—The intervention products were significantly more effective in reducing lesions than the placebo —The effect of the reference treatment was gradual over the 9 days, being more pronounced in the later part of the treatment period —A burning sensation was the most reported adverse event within the intervention group —In the placebo group no adverse events were reported	—Short duration of study and follow-up time —Small sample size in the different treatment groups —No demographics reported
Orafidiya et al. (2004; Nigeria) [51]	Preliminary clinical investigation (84; 12 subjects per product)	*Ocimum gratissimum* leaf essential oil preparations (lotion in 25% and 50% of aqueous aloe gel or 100% of aloe gel) (48)	—Reference/positive control: 1% clindamycin solution (12) —Negative control: Neat aloe gel dispersed in 1% polysorbate 80 (12) —Placebo: 40% of arachis oil emulsion in 1% *v*/*v* polysorbate 80 (12)	—Number of comedones, papules, and pustules (acne lesion count) —Number of days taken to achieve a 50% reduction in lesion count —Skin sensitivity -—Patient acceptability —Monitoring ADRs ^3^	4 weeks (baseline and weekly)	—In the negative control group, minimal activity was observed —A greater reduction in the substant lesion counts was observed in the intervention group compared to the negative and positive control groups —The preparations containing a greater quantity of aloe vera showed greater improved outcomes —Stinging sensation on the skin was the most reported adverse event (96%), which was tolerable —Only mild skin irritation was reported within the intervention group	—Short duration of study and follow-up time —Small sample size in the different treatment groups —No demographics reported
Hassoun et al. (2016; United States) [52]	Case study (1)	Cedarwood oil tretinoin cream (1)	NA	NA	4 weeks (baseline and 4 weeks)	—After 4 weeks of treatment, the patients’ faces were clear and had rare open and closed comedones on the chest with no papules	—Lack of comparison to a control, placebo, or standard therapy —Combination preparation —Only one case —Retrospective study —Uncontrolled

^1^ TTO: tea tree oil. ^2^ ASI: acne severity index. ^3^ ADRs: adverse drug reactions. ^4^ TLC: total lesion count. ^5^ TEWL: transepidermal water loss. ^6^ VAS: visual analog scale. ^7^ IGA: Investigator’s Static Global Assessment. Clinical trial registration numbers: Ref. [37]: NCT01930565 and Ref. [42]: NCT 01657110. NA = not applicable.

**Table 2:** Characteristics of the products used in the included studies focusing on acne. Details about the composition of the preparations are given in Table S1 (Supplementary Materials).

Author(s) (Publication Year; Country)	Intervention (Origin)	Intervention: Self-Made or Purchased	Control	Control: Self-Made or Purchased	Analyses of the EO-Containing Product
Enshaieh et al. (2007; Iran) [36]	5% TTO ^1^ gel (*Melaleuca alternifolia*)	Purchased from Cinere Company	Carbomer gel	Purchased from Cinere Company	The component levels were established through gas chromatographic analysis in accordance with the international standard
Kwon et al. (2014; South Korea) [37]	5% *Lactobacillus*-fermented *Chamaecyparis obtusa* (LFCO) cream (fermentation of *C. obtusa* by *Lactobacillus fermentum*)	Manufactured and provided by BST Corporation	5% TTO in cream	Manufactured and provided by BST Corporation	—Bacterial strains and MIC ^2^ determination —RT-PCR ^3^ —Ultra-performance liquid chromatography and high-resolution mass spectrometry analysis
Da Silva et al. (2012; Brazil) [38]	1% copaiba essential oil (*Copaifera langsdorfii*) in natrozol gel	Self-extracted and self-made	Natrozol gel	Purchased	—Density and phytochemical profile —High-resolution gas chromatography analysis for identification of the EO ^4^ components
Bassett et al. (1990; Australia) [39]	5% TTO ^1^ gel (*Melaleuca alternifolia* tree)	Purchased	5% benzoyl peroxide lotion	Purchased	Not conducted
Ghovvati et al. (2019; Iran) [40]	0.5% cinnamon gel (*Cinnamomum verum* bark oil)	Self-conducted extraction and preparation of topical gel	NA	NA	—Evaluation of color, odor, consistency, homogeneity, pH, density, and viscosity —Chemical quantification of the formulation
Talebi et al. (2020; Iran) [41]	1% (*w*/*w*) ajwain gel (*Trachyspermum ammi* (ajwain fruits)	Self-extracted and self-made	NA	NA	—Physicochemical evaluation: stability and viscosity —Gas chromatography analysis for identification of the EO ^4^ components
Malhi et al. (2017; Australia) [42]	200 mg/g TTO ^1^ gel and 7 mg/g tea tree face wash (*Melaleuca alternifolia*)	Purchased from Thursday Plantation	NA	NA	—Visual in vitro evaluation of MIC ^2^ after 48 h of anaerobic incubation (repeated thrice)
Hassan et al. (2013; Saudi Arabia) [43]	Eucalyptus oil (*Eucalyptus amaldulensis*) in cream	Self-mixed all the purchased constituents	NA	NA	Not conducted
Najafi-Taher et al. (2022; Iran) [44]	TTO ^1^ nano-emulsion (*Melaleuca alternifolia*) containing adapalene gel	Self-made adapalene-loaded TTO ^1^ nano-emulsion	Adapalene gel	Purchased from Aburaihan pharmaceutical Co.	—Characterization of nano-emulsion: DLS ^5^ and zeta potential analysis, TEM ^6^, viscosity, pH, and stability —In vitro: antimicrobial activity —Ex vivo: drug permeation and skin distribution —In vivo: skin irritation, systemic absorption, biochemical parameters, and histopathological analysis
Mazzarello et al. (2018; Italy) [46]	—Group A: 20% propolis, 3% TTO ^1^, and 10% *Aloe vera* (enzymatic hydrolysis of the original plant glycosides, *Melaleuca alternifolia* and *Aloe vera*)	Purchased	—Group B: erythromycin cream (positive control) —Group C: vehicle (placebo)	Purchased by Humana Pharma International Spa	Not conducted
Mazzarello et al. (2020; Italy) [47]	3.74% *Myrtus communis* oil, 0.1% *Origanum vulgare* oil, and 0.025% tretinoin (MOTC)	Self-made	1% clindamycin and 0.025% tretinoin (CTG)	Purchased	—Stability —pH —PET ^7^ (challenge test) —Susceptible test to different antibiotics
Parveen et al. (2009; India) [48]	Unani herbomineral cream	Self-made	Vehicle cream	Not mentioned	Not conducted
Kim and Shin (2013; South Korea) [49]	A basic, weekly acne treatment and a dermal application of a mixture of 3% TTO ^1^, 2%lavender oil, and jojoba oil	Unknown (the EOs are permitted by the Korean Food & Drug Administration)	Basic, weekly acne treatment.	Unknown (the acne intervention program was created through collaboration with a dermatologist, a member of the family practice faculty, and an aromatherapist)	Not conducted
Orafidiya et al. (2002; Nigeria) [50]	*Ocimum gratissimum* leaf EO preparations	Purchased	—Reference/positive control: 1% benzoyl peroxide solution	Purchased	Not conducted
Orafidiya et al. (2004; Nigeria) [51]	*Ocimum gratissimum* leaf EO preparations	Purchased	—Reference/positive control: 1% clindamycin solution	Purchased	Not conducted
Hassoun et al. (2016; United States) [52]	Cedarwood oil (from *Juniperus virginiana*) mixed with tretinoin cream	Cedarwood oil was purchased and self-added to the tretinoin cream by the patient	Not conducted	NA	NA

^1^ TTO: tea tree oil. ^2^ MIC: minimum inhibitory concentration. ^3^ RT-PCR: reverse transcription polymerase chain reaction. ^4^ EO: essential oil. ^5^ DLS: dynamic light scattering. ^6^ TEM: transmission electron microscopy. ^7^ PET: preservative efficacy testing. NA = not applicable.

### 2.3. Dermatitis and Eczema

Table 3 gives an overview of the characteristics, main results, and limitations of the included studies focusing on dermatitis and eczema. Table 4 gives an overview of the characteristics of the products used in the included studies focusing on dermatitis and eczema to assess their quality. The studies cover a range of interventions, with TTO, kānuka oil, and mixtures of EOs. The key findings of two case reports include the effectiveness of a mix of several EOs with a base shampoo to treat scalp dermatitis. The TTO gel and kānuka oil cream showed promising results in the subsequent treatment of facial seborrheic dermatitis and eczema, with minimal ADRs. The studies are discussed in order from the highest to lowest power of clinical evidence. 

Behehsti Roy et al. aimed to assess the effectiveness of TTO gel in treating mild-to-moderate facial seborrheic dermatitis. The study used a double-blind design with block randomization for treatment allocation involving 54 patients. The results showed a relatively increased improvement in the TTO group, with 39% achieving a total cure after two weeks. No allergic reactions or inflammation were reported. The control and vehicle products were provided by Dr. Jahangir’s Company, and the constituents were described in detail without additional analyses [53].

Shortt et al. investigated the use of 3% kānuka oil in an emollient cream base in treating moderate-to-severe eczema with a single-blind randomized controlled trial involving 80 participants. The results showed a significant improvement in the kānuka oil group, as evidenced by lower patient-oriented eczema measure (POEM) scores and a higher proportion of responders. The POEM score is a validated assessment measure derived from patient input and is used for monitoring the severity of atopic eczema. Furthermore, no significant differences in quality of life or safety issues were observed between the groups. In the kānuka oil group, 22 patients (53.7%) reported ADRs versus 15 (38.5%) in the control group. Three ADRs, including one case of transient stinging and two cases of exacerbated eczema, were determined to be related in the kānuka oil group. Two ADRs, both cases of exacerbated eczema, were reported in the vehicle control group. Hikurangi Bioactives Limited Partnership extracted the kānuka oil and Zealand Health Manufacturing compounded it into the study products. Both creams were produced following good nutraceutical manufacturing processes. No additional analyses were conducted, and the constituents were not reported [20]. 

De Valois reported a case study of a 72-year-old woman with severe scalp eczema experiencing extreme dryness, flaking, and itching. The initial treatments with olive oil and an antifungal shampoo (Nizoral, containing ketoconazole) were ineffective. An EO formula, including TTO, was applied as a shampoo and scalp soak. Over time, her condition improved considerably, with a complete clearance after seven months of daily treatment. The patient’s hair grew back, and her scalp returned to a healthy state. No ADRs were reported. The patient herself mixed the purchased EO formulation with the base shampoo and scalp soak [54].

Allan reported a case study of a 38-year-old man with long-standing seborrheic dermatitis on his scalp and face. The conventional medications with hydrocortisone creams provided temporary relief, but the condition returned upon discontinuation. The patient tried anti-dandruff shampoos with limited success. An EO mixture, including geranium, palmarosa, laurel, tansy, and spike lavender, was applied in a shampoo and a cream. Within a few days, the dermatitis disappeared, and the scalp remained clear for several months with some occasional, minimal reoccurrence on the face. Furthermore, no ADRs were reported. A base shampoo and cream were purchased after which the patient mixed them with the EO mixture [55].

### 2.4. Psoriasis

Table 5 gives an overview of the characteristics, main results, and limitations of the included studies focusing on psoriasis. Table 6 gives an overview of the characteristics of the products used in the included studies focusing on psoriasis to assess their quality. The studies cover a range of interventions, with frankincense oil (from *Boswellia* spp.), Soratinex^®^ products, ChP oleogel, and kānuka oil (from *Kunzea ericoides*). The key findings include improved Psoriasis Area and Severity Index (PASI) scores and other primary outcomes after the treatment with a *Boswellia*-based cream, Soratinex^®^, and ChP oleogel with a generally mild ADR profile. The studies are discussed in order from the highest to lowest power of clinical evidence. 

The double-blind, randomized study conducted by Thomas et al. explored the efficacy of kānuka oil-containing formulations compared to a control formulation for treating psoriasis. The study involved 30 individuals with psoriasis covering up to 10% of the body surface. The patients applied either the test formulation (with kānuka oil) or the control formulation (without kānuka oil) to the affected areas twice daily for 8 weeks. The primary endpoint was the change in the PASI score, and the secondary endpoint was the improvement in pruritus. Both formulations showed significant improvements in the signs of psoriasis and pruritus, with no statistically significant difference between the test and control formulations. Only minor ADRs were observed, including itchiness, after applying the scalp lotion (four subjects; 15.2%). The preparation of the intervention and control products was outsourced, and no additional analyses were conducted [56].

Fadaei et al. evaluated the safety and efficacy of the intervention consisting of a cream combining frankincense oil and ethanolic extract from *Boswelia* spp., with bioactive flavonoids from a licorice root extract (*Glycyrrhiza glabra* aqueous extract) and pumpkin seed oil (*Cucurbita pepo*), while the control group received the vehicle cream for the treatment of plaque psoriasis. A randomized, triple-blind, vehicle-controlled study with 108 participants was conducted. After 4 weeks, the *Boswellia*-based cream group had notable improvements in the scores for the psoriasis area and severity index (PASI), dermatology life quality index (DLQI), body surface area (BSA), and physician’s global assessment (PGA) compared to the control group. A significant portion of patients experienced PASI 50 and PASI 75 improvements. In the intervention group, two patients (4.2%) developed skin reactions including rash and severe itching and were withdrawn from the study. No other ADRs were reported. The researchers prepared both the intervention and control products with materials purchased from a local market in Teheran (Iran). Additionally, extensive analyses were conducted including the determination of the pH and the mechanical stability and microbial tests. Lastly, the constituents of each product were described in detail [33]. 

Kolahdooz et al. evaluated the efficacy of a topical herbal preparation called ChP oleogel on plaque psoriasis. ChP oleogel contains chamomile (*Matricaria recutita*) oil, pumpkin seed oil (*Cucurbita pepo*), and colloidal silicon dioxide. A randomized, intra-patient, double-blind, placebo-controlled trial with 40 patients was conducted. The ChP oleogel demonstrated significant improvements in the PASI, PGA, and patient satisfaction scores compared to the placebo, with minimal ADRs. Three patients (7.5%) reported itching and irritations, which were more severe on the side treated with the ChP oleogel. The intervention and control products were extracted and prepared by the researchers without conducting additional analyses [31]. 

Hercegova et al. investigated and described the efficacy and safety of Soratinex^®^ skincare products for the topical treatment of stable chronic plaque psoriasis in a non-controlled pilot study including 286 patients. After 8 weeks, a substantial number of patients had moderate-to-outstanding improvements, with some achieving a complete clearance or significant reductions in lesions. Temporary ADRs were reported including folliculitis (fifteen subjects; 6.0%) and contact dermatitis (seven subjects; 28%). The Soratinex^®^ products were purchased, and no analyses were conducted, whereas the constituents were described in detail [57].

Fioranelli et al. investigated the efficacy and safety of Soratinex^®^ skincare products in treating chronic plaque psoriasis in a non-controlled pilot study including 30 patients with mild-to-moderate psoriasis vulgaris. Five patients showed no clinical improvement, six had slight improvements, nine had a good response, and five showed an excellent improvement. Mild and transient itching without redness of the skin was reported (26.9%). Furthermore, one patient (3.8%) with scalp psoriasis dropped out due to the development of a folliculitis-like inflammation. The Soratinex^®^ products were purchased, and no analyses were conducted, whereas the constituents were described in detail [58]. 

### 2.5. Rosacea

Table 7 gives an overview of the characteristics, main results, and limitations of the included studies focusing on rosacea. Table 8 gives an overview of the characteristics of the products used in the included studies focusing on rosacea to assess their quality.

The study conducted by Ebneyamin et al. aimed to evaluate the effectiveness and safety of a topical gel containing 2.5% of permethrin with TTO (no concentration mentioned) versus a placebo (with a carbomer gel base) in reducing *Demodex* mites and improving clinical symptoms in individuals with rosacea. The study was a randomized, double-blind, placebo-controlled clinical trial and included 47 patients with mild-to-moderate papulopustular rosacea. After five weeks, the test product was more effective than the placebo in reducing the mite density, and, after twelve weeks, the treatment side showed a significant reduction in mite density, as well as an improvement in the papules, pustules, burning, stinging, and dryness. However, no significant changes were observed in plaques, telangiectasia, flushing, or edema. The global assessments by patients and physicians both indicated a better improvement in the permethrin with the TTO group compared to the placebo group. The ADRs included burning, stinging, dryness, erosion, erythema, itching, scaling, and vesiculation, with no major allergic reactions. Nothing was reported about the origin of the intervention and control products, and no additional analyses were conducted [58].

## 3. Discussion

### 3.1. General Aspects

There is a growing interest in the use of topical treatments that are effective, easy to use, and generally have minor ADRs [1]. Therefore, this scoping review provides an extensive overview of the current knowledge regarding the efficacy, safety, product characteristics, and quality of topical treatments containing EOs used in a clinical setting to treat inflammatory skin conditions. The search strategy focused on the four most prevalent inflammatory skin conditions including acne, dermatitis and eczema, psoriasis, and rosacea. Overall, limited clinical studies on the application of EOs in the treatment of inflammatory skin conditions have been performed. Preclinical research on the inflammatory effects of EOs in in vitro models is more abundant [12], supporting the traditional use of EOs in the treatment of inflammatory skin processes and constructing a bridge to studies in humans. 

### 3.2. Tea Tree Oil (TTO)

TTO stands out as the most extensively examined EO from this scoping review. Nine studies investigated the efficacy of treatments with TTO alone or in combination with other constituents to treat acne, dermatitis and eczema, psoriasis, and/or rosacea [36,39,42,44,46,47,49,53,54,59]. TTO is obtained by the steam distillation of the leaves and branches of *Melaleuca alternifolia*, a tree belonging to the Myrtaceae family [7]. The main component of TTO is terpinene-4-ol. *M. alternifolia* exhibits distinct oil chemotypes characterized by varying percentages of terpinen-4-ol, 1,8-cineole, and terpinolene [60]. The in vitro anti-inflammatory, antibacterial, antiviral, and antifungal properties of TTO make it an interesting treatment option for inflammatory skin conditions [12,60]. The antibacterial, antiviral, and antifungal properties have been ascribed to terpinene-4-ol, whereas the anti-inflammatory effect has been attributed to other terpenoids [60]. 

Comparing the acne-related assessment outcomes after the use of 5% TTO gel with 5% of benzoyl peroxide, a better symptom reduction was found with benzoyl peroxide by comparison [38]. Nevertheless, the TTO gel had substantially fewer ADRs compared to the 5% benzoyl peroxide, and the TTO gel significantly reduced acne-related assessment outcomes compared to the placebo [36,39]. The efficacy of a 5% TTO gel has also been reported for the treatment of facial seborrheic dermatitis by significantly reducing the scores for itching, erythema, scaling, and greasy crusts and the scoring of patient satisfaction, compared to a placebo group and compared to the baseline values [53]. Additionally, a 20% TTO gel and a 0.7% TTO face wash showed a significant reduction in the total lesion count (TLC) and Investigator’s Static Global Assessment (IGA) score after 8 weeks, compared to the baseline, demonstrating the efficacy of TTO to treat acne [42]. 

Furthermore, this present scoping review showed the potential of TTO to enhance the effects of conventional acne treatments such as adapalene gel [44]. This study showed a relatively better improvement in the number of (non-)inflammatory lesions, TLC, and ASI after treatment with a TTO nano-emulsion containing adapalene gel compared to a marketed adapalene gel. Additionally, a combination product containing tea tree, lavender, and jojoba oil was tested to treat acne and a 2.5% permethrin with TTO gel to treat rosacea [49,59]. It remains a challenge however, to differentiate the individual contribution of each constituent in a combination product. The absence in the study design of a focus on the separate effects of each constituent makes it unclear how TTO specifically influences the observed therapeutic effects and ADRs. 

Based on the combined results, it seems plausible that patients suffering from ADRs from the standard acne treatment may consider switching to a 5% TTO gel, which is still effective but has fewer ADRs. Additionally, the individuals who experience poor results with a conventional acne treatment may consider enhancing their treatment by combining it with TTO to achieve better outcomes and acceptability. 

### 3.3. Placebo-Controlled Clinical Studies 

Six studies were retrieved in this scoping review that solely compared the intervention to a placebo (vehicle) [20,31,33,38,48,56]. Placebo controls are interventions employed in clinical studies that lack the active components of the experimental treatment [61]. Placebos are considered instruments, serving as research tools that help discover the genuine effectiveness and mode of action of ‘authentic’ medications [62]. The objective of using placebo control interventions is to establish a baseline for evaluating the efficacy of the active interventions, allowing a comparison of their effects [61]. The goal is to have an equitable assessment of the drug’s superiority compared to a placebo and of the drug’s non-inferiority when compared to another drug [62]. The use of a placebo control product strengthens a clinical trial.

Three studies prepared an EO and other constituents in a hydrophilic cream base [20,33,48]. Although the constituents of the cream base varied between studies, the emollient and moisturizing properties remained consistent. Moreover, an indifferent cream base itself is not expected to elicit ADRs, which makes the safety assessment of the active treatment valid, because any ADRs can be principally attributed to an active ingredient. However, Shortt et al. reported as many as fifteen ADRs after treatment with the placebo cream base in their trial investigating the efficacy of kānuka oil in the treatment of moderate-to-severe eczema, with 80 participants [20]. Since no information regarding the components of the cream base was provided, the cause for the observed ADRs remains undetermined. Shortt et al. attributed the improvement of the disease state in the placebo group to the emollient and moisturizing properties of the cream base. Furthermore, the restoration of the hydration state of the skin to normal was attributed to the cream base [20]. 

Regarding the effectiveness of the aforementioned EOs in combination with the cream base [20,33,48], a randomized controlled trial using 3% kānuka oil cream to treat eczema showed no significant difference in the Patient Oriented SCOring Atopic Dermatitis (PO-SCORAD), Dermatology Life Quality Index (DLQI), and Treatment Satisfaction Questionnaire for Medication (TSQM) between the intervention and placebo group suggesting that the effect of the 3% kānuka oil was restricted to diminishing the frequency of symptoms, rather than reducing their overall severity [19]. On the contrary, the Unani herbomineral cream (UHC) significantly improved the IGA in acne patients [48].

Additionally, a previous placebo-controlled, double-blind, randomized trial showed that a topical cream based on *Boswellia serrata* oil (frankincense) and an ethanolic extract of Boswellia was an effective anti-inflammatory solution for reducing scales and erythema in psoriatic patients [63]. The oleogum resin, obtained by tapping *Boswellia* trees, is steam distilled, yielding the EO, which is also known as frankincense oil or olibanum. Typically, α-pinene, α-phellandrene, limonene, β-myrcene, β-pinene, p-cymene, and β-caryophyllene are the main constituents [10]. The promising results of a topical cream based on *Boswellia serrata* oil aligns with the findings of a subsequent clinical trial, where a ≥50% reduction in the PASI score was seen in 53.19% of patients, and a ≥75% reduction in the PASI score was noted in 21.28% of the patients after using the *Boswellia* cream [33]. The frankincense properties can be linked to the active biological compounds of the *Boswellia* cream, including boswellic acids, diterpenoids, and triterpenoids (contained in the ethanolic extract). Furthermore, *Boswellia* cream contained pumpkin oil possessing antiseptic, anti-eczematous, antidermatitic, antitoxic, and antipyretic properties [33]. Boswellic acid has anti-inflammatory properties, but would not be present in the EO because its molecular mass is relatively high.

Another randomized clinical trial retrieved from the search strategy showed the positive effects of a combination of chamomile (*Matricaria chamomilla*) oil with pumpkin (*Cucurbita pepo*) seed oil. A significant enhancement in various psoriasis-related factors was found in comparison to the placebo group, which was treated with liquid paraffin [31]. Bisabolol and chamazulene, the main components of the chamomile EO, possess anti-inflammatory properties [3]. 

Additionally, two other studies comparing an EO product with a vehicle to investigate the efficacy and safety of the EO product, used natrozol gel and ointment/lotion to treat acne and psoriasis, respectively [38,56]. A highly significant decrease in the extent of the area affected by acne in both the intervention group treated with 1% copaiba EO in natrozol gel and the placebo group treated with only natrozol gel was observed showing the potential to treat mild acne [38]. Copaiba oil is obtained via the hydrodistillation of copaiba balsam, an oleoresin from *Copaifera officinalis*. The EO contains many sesquiterpenes, among which β-caryophyllene is the most abundant [10]. In contrast, the kānuka oil-containing ointment and lotion showed no significant differences after treatment when comparing the control and treatment groups. This aligns with previous studies that have concluded that there is no compelling reason to consider the kānuka oil as an alternative for psoriasis treatment [56]. Kānuka oil comes from the leaves and twigs of *Kunzea ericoides*. It contains monoterpenes with α-pinene, 1,8-cineole, and α-terpineol as main components [10].

### 3.4. Clinical Studies without Comparison to a Control, Placebo, or Standard Therapy

In this scoping review, seven studies were identified that conducted research without comparison with a control, placebo, or standard therapy, called uncontrolled studies/trials [40,41,43,52,55,57,58,64]. Uncontrolled studies are known for yielding higher mean effect estimates than controlled studies, potentially inflating expectations regarding the intervention. This design carries a risk of inherent bias, and its results are generally considered less reliable than those of randomized controlled studies. Additionally, using this design in cases of spontaneously resolving conditions can further exaggerate the perceived effect [64]. However, uncontrolled studies can give insights into the potential efficacy of a new drug compound. 

Of those seven uncontrolled studies, four were specifically centered on treating acne, each employing distinct products [40,41,43,52]. Analyzing the results of the uncontrolled studies, a clinical trial investigated the efficacy and safety of a 1% (*w*/*w*) ajwain gel showed improvements in the acne-related parameters without reported ADRs, possibly indicating the safety of the product. The observed effect can be assigned to the anti-inflammatory, antimicrobial, and anti-oxidant effects of *Trachyspermum ammi* fruits, the origin of ajwain (or ajowan) EO, described in in vitro investigations [41]. Ajwain EO contains p-cymene, γ-terpinene, thymol, and carvacrol as its main constituents [64]. Comparing the 1% ajwain gel with a 0.5% cinnamon bark gel, several distinctions could be observed [40,41]. Cinnamaldehyde is the main constituent of the EO from cinnamon bark [7]. The application of cinnamon gel led to a notable reduction in the size of the fluorescence spots, while ajwain gel was effective in decreasing both the size and the number of these spots. However, the alterations in the skin’s biophysical profile following the application of ajwain and cinnamon gel were comparable. Both formulations significantly reduced the non-inflammatory and inflammatory lesion counts, the sebum content, and the erythema index compared to the baseline. Regarding safety, the use of ajwain gel resulted in no (reported) ADRs, while thirteen patients reported mild burning and eleven patients reported facial redness after using the cinnamon gel. Additionally, another uncontrolled trial showed positive results in all patients after the second week of treatment with eucalyptus oil cream in the treatment of acne vulgaris [43]. Although the results of the uncontrolled trial look promising, the study design is unreliable. The trial included only five participants who were observed over ten days, while the primary and/or secondary outcomes were not defined, and no safety assessment was conducted. Comparable limitations exist for the case study that describes the additional value of cedarwood oil combined with tretinoin to treat acne [52]. Therefore, additional research is required to assess the potential of eucalyptus oil and cedarwood oil in the treatment of acne. 

Regarding the treatment of stable chronic plaque psoriasis with combination products including EOs (Soratinex^®^), two uncontrolled pilot studies were conducted in a Hungarian (251 patients) and Polish (30 patients) population [57,58]. Following the treatment period of 6 to 8 weeks, 46% of Hungarian patients and 17% of Polish patients showed a 75–100% reduction in the PASI scores. However, in both patient populations, mild and temporary itching was reported, with folliculitis proving to be a recurring ADR in both preliminary studies. The combination of different EOs in the Soratinex^®^ products makes it impossible to identify the contribution of individual EOs. Despite this obvious limitation, these studies may serve as the foundation for an extended investigation into these products for the treatment of stable chronic plaque psoriasis, which should involve comparisons with placebos and/or standard therapy, longer study durations, and a broader spectrum of endpoints or outcomes.

Finally, one case study showed the disappearance of the patients’ seborrheic dermatitis in a matter of days after being treated with shampoo and an aqueous-based cream containing an EO mixture [55]. While the combination of EOs demonstrates promising effects, a single case retrospectively examined is not conclusive. Therefore, further extensive research is required. 

### 3.5. Clinical Studies with Comparison to Standard Therapy

One study conducted a clinical trial comparing MOTC cream, which served as the intervention, and CTG cream, which acted as the positive control [47]. The MOTC cream contained two EOs: from myrtle (*Myrtus communis*) and from oregano (*Origanum vulgare*); the CTG cream contained 1% clindamycin. Additionally, both creams contained 0.025% tretinoin (a retinoid). Due to the absence of a placebo, this trial can only draw conclusions regarding the efficacy and safety of the intervention compared to the already established treatment (the positive control). Overall, both products improved the clinical parameters at each assessment time, and no statistically significant differences were observed in the inter-group comparison. The MOTC cream proved significantly more effective than the CTG in reducing the papular erythema index. Furthermore, erythema in the healthy inter-papular skin area increased significantly only in the skin treated with the CTG. In conclusion, this clinical trial suggests that MOTC cream holds promise as a potential alternative to antibiotics in formulations with retinoids. However, determining the specific contributions of the two EOs versus the low concentration of tretinoin, or any potential synergistic effects, remains challenging. Furthermore, there were no reference data available because of the absence of a placebo, and the clinical trial’s reliance on a small sample size amplified the inter-individual variability.

### 3.6. Clinical Studies with Comparison to a Placebo, Positive Control, and Negative Control 

Orafidiya et al. conducted two consecutive clinical studies [50,51]. The first trial aimed to clinically establish the ideal concentration of African basil (*Ocimum gratissimum*) oil and the most suitable base for the effective treatment of acne vulgaris, comparing it with a reference drug product (clindamycin) and a placebo. The second trial aimed to assess the impact of *Aloe vera* leaf gel on the anti-acne properties of ocimum oil, as well as to compare the effectiveness of both agents individually and in combination with a positive control (1% clindamycin solution), a negative control (neat aloe gel dispersed in 1% polysorbate 80), and a placebo. A negative control outcome is an outcome that exhibits similar potential sources of bias as the primary outcome but lacks a plausible connection to the treatment under investigation. Adding a negative control to the clinical trial serves as an approach to detect and reduce bias [65]. Preparations containing ocimum oil, particularly at concentrations of 2% and 5% in alcohol and in cetomacrogol blend bases, have demonstrated a superior effectiveness and a quicker reduction in lesion counts compared to the positive control. Additionally, a favorable effect of *Aloe vera* leaf gel was found by observing increasing improvements in acne lesions with increasing *Aloe vera* content in the preparations. 

### 3.7. Reported ADRs

Overall, the clinical studies revealed that ADRs were more frequently reported in the group receiving the EO-containing intervention than in the placebo group. However, the number of reported ADRs was higher in patients receiving the standardized (conventional) treatment compared to the EO-containing intervention group. The most reported ADRs, after using an EO-containing gel to treat the acne or rosacea, were dryness of the skin, mild burning, and erythema, which typically disappeared within 30 min after the application [36,37,40,44,50,51]. The clinical studies involving EO-containing products treating psoriasis primarily reported itching and folliculitis as ADRs [31,56,57,58]. Notably, nine out of the thirty-one studies, including five case studies, did not monitor nor record ADRs, creating a knowledge gap regarding the product’s tolerability [38,42,43,46,47,48,49,52,54,55]. Furthermore, a few studies mentioned that only minor or no serious ADRs were reported without specifying details [20,33,41,53]. The studies involving TTO products reported ADRs consistent with those reported for the EO products, including dryness, erythema, and a burning sensation [37,39,44,59]. No severe ADRs were reported, and only a small number of patients withdrew from the trial due to ADRs without any improvement of the disease. In conclusion, the EO-containing treatment comes with ADRs, but there is an improvement in tolerability compared to standardized treatments. 

### 3.8. The Quality of the EO Products

Concerning the quality of the EO products, most clinical studies acquired the EO products from commercial sources, while no further quality evaluation analyses were performed [20,36,39,40,43,44,49,50,51,52,54,57,58,59]. Therefore, the identity and quality of these products has not been established and cannot be guaranteed. Nonetheless, it is possible that the authors obtained quality assessments from the manufacturers; however, these were not mentioned. A comparison of the outcomes of repeated studies is therefore difficult or even impossible. Often the authors stated from which manufacturer the EO products were purchased, allowing the possibility of research depending on the transparency of the specific manufacturer. In contrast, six clinical studies prepared the EO products themselves and conducted several analyses to identify the substituents and to control quality and stability [33,38,40,41,44,47]. Additionally, two clinical studies acquired their EO products externally but conducted independent analyses [37,56]. The execution of the analyses with concomitant transparent documentation yields a higher quality of study, because the EO products are well described. Furthermore, in several clinical studies, the EO products were prepared by the investigators, but they did not carry out any subsequent analyses, resulting in uncertainties related to the quality of the final products [31,43,48,55]. 

What is important for preclinical and clinical research on herbal products and for their use in practice and patient care is their characterization, as well as the quality validation. Unfortunately, these aspects are regularly neglected or underestimated. Missing details about the source material, preparation method, the chemical composition of a product (qualitative and quantitative), its principal constituents, contents, and possible standardization should be considered as a major limitation in the scientific communication, as well as in conveying information about a product to the healthcare provider and the user [3,5,6,9,10,66]. 

Most EOs are obtained by the hydrodistillation of fresh or dried plant material. This implies that only volatile constituents are found in the product, with limits regarding molecular mass and polarity. The EOs obtained by expression (e.g., from *Citrus* fruits) may contain (furano)coumarines. Also available are extracts produced with non-polar solvents like n-hexane. These products may be marketed as such but are not real EOs (according to the definition). They are actually of a lower quality and are generally cheaper. In general, the production method will strongly influence the composition and quality of the product. In addition, the place where the EO is produced and where the plants were growing (the latitude, soil, sunlight, and rainfall) affect the composition of the EO [3,5,6,9,10,66].

It is very important to know exactly which source and plant species (and part) was used to produce the EO. Possible adulterations and contaminations must be identified to avoid fraud and low quality, as well as toxic products. The identity of the plant species must be determined and confirmed by professionals. This leads to the correct Latin binomial species name and author. Synonyms and homonyms easily lead to confusion. Finally, mistakes with the source plant may possibly lead to the occurrence of ADRs during use [3,5,6,9,10,66]. 

The chemical composition of an EO must be reported, at least regarding the main constituents and their contents, as this usually relates to the safety and bioactivity. Gas chromatography (CG-FID) and gas chromatography coupled with mass spectrometry (GC-MS) are common analytical techniques to determine this, combined with a reference library [3,5,6,9,10,66].

For a number of pharmaceutically used EOs, more than one species is allowed according to the monographs of the European Pharmacopoeia (Ph. Eur.) and the European Medicines Agency (EMA) [67]. For instance, Eucalyptus oil (Eucalypti aetheroleum) for application in the therapeutic areas of pain and inflammation and coughs and the cold can be obtained from *Eucalyptus globulus* Labill., *Eucalyptus polybractea* R.T. Baker, and *Eucalyptus smithii* R.T. Baker. 

Intraspecies variation may lead to differences in the composition of EOs. Examples of the potential causes for fluctuations are environmental, including the growing conditions, and the development stage of the plant at the time of harvesting. The Ph. Eur. sets the upper and lower limits for a number of the main constituents for EOs, of which a monograph is included. Furthermore, several species of chemotypes exist (e.g., *Thymus vulgaris* ct. geraniol, linalool, thujanol-4, and carvacrol are distinguished), yielding considerably different EO compositions, both regarding the principal constituents and contents. Concomitantly, this may result in different profiles of biological activities [3,5,6,9,10,66]. 

Finally, analytical data should prove that the EOs were stored properly and that the shelf life was not exceeded. The decomposition of the EO constituents, which are often oxidation products (especially if the EO constituents contain double bonds) under the influence of elevated temperatures and light has been described in the literature [8]. This leads to the inferior quality of a product upon aging and may be the cause of ADRs associated with their use. A well-known example is oxidation products of terpinene-4-ol in improperly stored or old tea tree oil [68].

To guarantee the quality and associated biological activity and safety, it is preferable that pharmaceutical grade EOs (complying to pharmacopoeia requirements) should be used for therapeutic purposes. 

### 3.9. Limitations of the Clinical Studies

As already mentioned, some studies lack the comparison with a placebo and/or standardized treatment. Additionally, in three clinical studies, the patients had to apply the intervention and placebo on two different parts of the body suffering from the disease [31,37,59]. This approach may increase the risk of cross-contamination and confusing the tubes. The advantage is that inter-individual differences are ruled out. Only five clinical studies included 100 or more eligible participants [33,39,44,50,57]. An appropriate large sample size of a clinical trial is necessary to give it statistical power and to detect clinically relevant differences between the intervention and placebo groups. A clinical trial involving a limited number of participants is more susceptible to variability and bears a significant risk of failing to show the effectiveness of a given intervention even when it is genuinely present [69]. Moreover, the duration of the clinical studies ranged from 10 days to 7 months, with the majority lasting between 4 and 8 weeks. A short study duration has the consequence of not examining the longer-term effects and ADRs. Additionally, when an intervention, a placebo, or a positive control requires more time to elicit effects, this may remain unnoticed. On the other hand, shorter trial durations can enhance patient participation, and one could argue whether an intervention that does not yield significant improvements within a few weeks is suitable for clinical practice. The time needed to see an effect of the treatment also concurs with the nature of the ailment that is treated. Finally, for only two studies [37,42] clinical trial registration numbers were retrieved from ClinicalTrials.gov. Although (pre)registration of clinical trials should be common practice nowadays, providing access to information that can be used for further analyses [70], it is known that a substantial portion of trials are published without a registration number [71].

### 3.10. Strengths and Limitations of this Scoping Review

The main strength of this scoping review is the comprehensive broad overview of the conducted clinical studies with topical treatments containing EOs used for inflammatory skin disease, together with a critical assessment of the information about the characteristics, quality, efficacy, and safety of the EO products used. This targeted approach highlights knowledge gaps and opportunities for further studies, including the need for more research identifying the EO product constituents or enhancing transparency in their reporting. The need for clinical studies with larger sample sizes and extended study durations has become clear from our scoping review. Our work also has several limitations. For example, four eligible articles were excluded because the authors of the current review only had access to a congress abstract providing very limited data. Nonetheless, this information could have provided further insights into the comprehensive picture of EO products. Finally, the data quality obtained from the literature and the databases utilized in this scoping review could be compromised by inconsistencies due to the absence of critical information, such as the origin and quality of the EOs. 

### 3.11. Future Perspectives

This scoping review yielded insights into the future prospects and limitations regarding the utilization of EO-containing products for treating acne, dermatitis and eczema, psoriasis, and/or rosacea. In addition, the protocols of three clinical studies were found to be registered in the Iranian Registry of Clinical Trials [72,73,74], but the results have not (yet) been published. Taken together, the findings so far indicate that good quality research on pharmaceutical grade EO-containing products remains valuable, with promising results anticipated. 

## 4. Materials and Methods

### 4.1. Search Strategy

This scoping review was performed following the Preferred Reporting Items for Systematic Reviews and Meta-Analyses (PRISMA) Extensions for Scoping Reviews (PRISMA-Scr) [34]. A systematic search was conducted by using four global electronic databases, PubMed, Embase, the Cochrane Library, and Scopus, from 28 September to 13 October 2023. Clinical studies and case reports investigating the efficacy and safety of EOs used to treat acne, dermatitis and eczema, psoriasis, and/or rosacea were included. Only articles written in Dutch or English, without any publication date limits, were included. The search strategies, individualized for each electronic database, used are provided in Table S2 (Supplementary Materials). This study was registered at the Open Science Framework (OSF), https://osf.io, on 25 January 2024. 

### 4.2. Study Selection

Initially, the title and abstract were screened and studies were considered to be eligible for inclusion if they met one of the following inclusion criteria:

1. Clinical studies, case studies, and randomized controlled studies making use of a product containing an EO or multiple EOs to treat acne, dermatitis and eczema, psoriasis, and/or rosacea.
2. Reported efficacy and safety outcomes after the use of the intervention, control, and/or placebo.

Studies were excluded if they met any of the following exclusion criteria:

1. The studies were conducted with in vitro or in vivo models only.
2. The article was a review or clinical trial protocol.
3. The article’s full text was not available.
4. Articles that reported an inflammatory skin condition caused by the use of a product containing an EO.
5. Articles that made use of fixed plant-derived oils (triacyl glycerides) only.

### 4.3. Data Extraction

The articles were selected and duplicates were removed using the EndNote20 software [75]. Upon the inclusion of the studies in this study, the data were extracted into a Microsoft Word and Excel file. The extracted study characteristics of interest included the following: the study objective, study design, type of intervention, control and/or placebo, number of participants and their demographics, indication, concentration and dosage of the products, primary and secondary outcomes, efficacy and safety results reported, intervention and control constituents, origin, effects, preparation and self-made or purchased, and additional analyses conducted on the intervention and control products. 

The data extraction was conducted by a single researcher (A.E.W.K.D.). To decrease the potential for errors and the risk of missing relevant clinical studies, a randomly taken sample of ten included and ten excluded articles was assessed by another researcher (C.E.). 

## 5. Conclusions

This scoping review provides a broad overview of the current knowledge regarding the product characteristics, quality, efficacy, and safety of topical treatments with EOs for inflammatory skin conditions in humans. Overall, the clinical evidence for the efficacy of EOs to treat these conditions is sparse and incommensurate. The clinical studies were classified as limited regarding the sample size and trial duration, while research on the longer-term effects in a broad population is required. Furthermore, most studies did not specify the characteristics of the EO products used, leaving a knowledge gap in the quality of the EO products and their conclusive mode of action. Thus, extended placebo-controlled clinical studies with larger groups of eligible patients and a longer study duration are necessary to assess the promising potential of EO products to treat inflammatory skin conditions. 

## Figures and Tables

**Table 3:** Characteristics, main results and limitations of the included studies focusing on dermatitis and eczema. Details about the composition of the preparations are given in Table S1 (Supplementary Materials).

Author(s) (Publication Year; Country)	Study Design (n)	Intervention (n)	Control (n)	Outcome Measure(s)	Study Duration (Evaluation Period)	Main Results	Limitations
Behehsti Roy et al. (2014; Iran) [53]	Randomized, vehicle-controlled, double-blind design (54)	5% TTO ^1^ gel (27)	Vehicle gel (27)	—Severity of seborrheic dermatitis —Extent of itching, erythema, scaling, and greasy crusts score —Patient satisfaction —Monitoring ADRs ^2^	4 weeks (baseline; 2 and 4 weeks)	—After 4 weeks, a significant decrease in the values of all parameters in the intervention group was observed, but there was no change in the vehicle group —After 4 weeks of treatment, 91% of the intervention group revealed a total cure —No allergic irritation or inflammation was reported	—Lack of comparison with standard therapy —Small sample size —Short duration
Shortt et al. (2022; New Zealand) [20]	Single-blind, parallel group, superiority, randomized controlled trial, in a community setting (80)	3% kānuka oil in cream (41)	Vehicle cream (39)	—POEM ^3^ —PO-SCORAD ^4^ —DLQI ^5^ —TSQM ^6^ —Photographs of eczema lesions —Monitoring ADRs ^2^, compliance, and concomitant medication use	6 weeks (baseline; 6 and 8 weeks)	—The mean POEM ^3^ showed a statistically significant improvement with a greater degree in the intervention group compared to the control group —More participants in the intervention group experienced a clinically significant improvement of their POEM ^3^ score compared to the control group —No statistical difference in the PO-SCORAD ^4^, DLQI ^5^, and TSQM ^6^ scores between groups was observed —22 ADRs ^2^ were reported by the intervention group compared to 15 in the control group (no serious ADRs ^2^)	—Odor of the intervention might lead to bias —Bias between pharmacists and dermatologists —Short duration
De Valois (2004; United Kingdom) [54]	Case study (1)	EO ^7^ formulation (base shampoo and scalp soak) (1)	NA	NA	7 months (baseline; 2, 3, and 7 months)	—At 2 months, the condition was improved considerably but had not disappeared —At 3 months, continued improvement was observed, but the condition was still lingering —At 7 months, the condition had completely disappeared	—Only one case —Retrospective study —Uncontrolled —Patient was non-adherent and was not applying the intervention as prescribed
Allan (2003; unknown) [55]	Case study (1)	Drops of EO ^7^ mixture (base shampoo) and a 2% dilution of the EO ^7^ mixture (aqueous-based cream) (1)	NA	NA	Number of weeks (few days, number of weeks, and number of months)	—Within a few days, the problem had completely gone —After stopping treatment for a number of months, the scalp had remained clear, but within the first weeks a tiny amount of dermatitis would reappear on his face —By using the cream once a week on his face, the areas affected by dermatitis remained clear	—Only one case —Retrospective study —Uncontrolled

^1^ TTO: tea tree oil. ^2^ ADRs: adverse drug reactions. ^3^ POEM: patient-oriented eczema measure. ^4^ PO-SCORAD: patient-oriented scoring atopic dermatitis. ^5^ DLQI: dermatology life quality index. ^6^ TSQM: treatment satisfaction questionnaire for medication. ^7^ EO: essential oil. NA = not applicable.

**Table 4:** Product characteristics of the products used in the included studies focusing on dermatitis and eczema. Details about the composition of the preparations are given in Table S1 (Supplementary Materials).

Author(s) (Publication Year; Country)	Intervention (Origin)	Intervention: Self-Made or Purchased	Control	Control: Self-Made or Purchased	Analyses of the EO-Containing Product
Behehsti Roy et al. (2014; Iran) [53]	5% TTO ^1^ gel (*Melaleuca alternifolia*)	Purchased from Dr. Jahangir’s Company	Vehicle gel	Purchased from Dr. Jahangir’s Company	Not conducted
Shortt et al. (2022; New Zealand) [20]	3% kānuka oil cream (*Kunzea ericoides*)	Purchased	Vehicle cream	Not mentioned	Not conducted
De Valois (2004; United Kingdom) [54]	EO ^2^ formulation with base shampoo and scalp soak	Purchased	NA	NA	Not conducted
Allan (2003; unknown) [55]	Drops of EO ^2^ mixture, base shampoo, and a 2% dilution of the EO ^2^ mixture with an aqueous-based cream origin	Patient mixed 2–3 mL base shampoo with four drops of the EO ^2^ mixture	NA	NA	Not conducted

^1^ TTO: tea tree oil. ^2^ EO: essential oil. NA = not applicable.

**Table 5:** Characteristics, main results, and limitations of the included studies focusing on psoriasis. Details about the composition of the preparations are given in Table S1 (Supplementary Materials).

Author(s) (Publication Year; Country)	Study Design (n)	Intervention (n)	Control (n)	Outcome Measure(s)	Study Duration (Evaluation Period)	Main Results	Limitations
Thomas et al. (2015; Australia) [56]	Randomized, active-controlled, double-blind design (30)	20% kānuka oil in ointment and/or scalp lotion (15)	The same as the test formulation, but without the kānuka oil component (15)	—PASI ^1^ —Pruritus (VAS ^2^ scoring) —Monitoring ADRs ^3^	8 weeks (baseline; 2, 4, and 8 weeks)	—Two individuals from the test group and three from the control group did not respond to the treatment —After 8 weeks, both treatments showed significant improvements and the PASI ^1^ and VAS ^2^ scores decreased equally in the test group and control group (no significant difference between the groups) —Individuals treated with the test scalp lotion displayed a statistically comparable improvement rate compared to the control scalp lotion —No serious adverse events were reported; only 4 out of 26 individuals that used the scalp lotion reported itchiness in the beginning of the treatment	—Short-term follow-up —Control medication containing coal tar and salicylic acid —Short duration of the study
Fadaei et al. (2021; Iran) [33]	Randomized, triple-blind, vehicle-controlled, parallel group study (108)	Frankincense oil (plus other constituents) in cream (45)	Vehicle cream (54)	—Photos of skin lesions —Compliance —PASI ^1^ —PGA ^4^ —BSA ^5^ —DLQI ^6^ —Pruritus severity scale —Patient satisfaction —Monitoring ADRs ^3^	4 weeks (baseline; 2 and 4 weeks)	—After 4 weeks of intervention, 53.19% of the patients in the intervention group and 2.44% in the vehicle group achieved a PASI ^1^ of 50% —Mean DLQI ^6^, PGA ^4^, and pruritus scores decreased in both groups over time —Mean BSA ^5^ score decreased significantly over time in the intervention group but not significantly in the vehicle group —After 4 weeks of intervention, the median patient satisfaction score was significantly higher in the intervention group compared to the vehicle group —2 patients reported skin reactions 24 h after exposure to the intervention	—Lack of comparison with a current topical treatment —Short duration of study and follow-up time
Kohladooz et al. (2018; Iran) [31]	Randomized, intra-patient, double-blind, placebo-controlled clinical trial (40)	Chamomile-pumpkin (ChP) in oleogel (40)	Vehicle (40; liquid paraffin)	—Erythema, scaling, and induration severity scores —PSI ^7^ —PGA ^4^ —Photographs of the lesions —Patient satisfaction —Monitoring ADRs ^3^	4 weeks (baseline and 4 weeks)	—After 4 weeks, the PSI ^7^ score decline was significantly greater in the intervention group compared to the placebo group —After 4 weeks, the PGA ^4^ and overall patient satisfaction scores showed a significantly greater improvement in the intervention group compared to the placebo group —3 patients reported itching and irritation, which was more severe in the intervention group	—Lack of comparison with a current topical treatment —Risk of cross-contamination —Short duration of study and follow-up time —Small sample size
Hercegova et al. (2016; Italy) [57]	Non-controlled pilot study (251)	Soratinex^®^ scalp and body cleansing gel; scalp and body ointment and skin conditioner (251)	NA	—PASI ^1^ —Photography analysis —Monitoring ADRs ^3^	8 weeks (2 weeks before treatment; baseline; 1, 2, 4, 6, and 8 weeks)	—After 8 weeks, 13 individuals had no clinical improvements, 46 had a moderate improvement, 77 showed a good improvement, and 115 patients had an outstanding improvement —ADRs ^3^ were mild and temporary; however, 15 patients reported folliculitis and 7 patients reported contact dermatitis	—Lack of control group —Short duration of study and follow-up time —Small amount of outcome measures
Fioranelli et al. (2016; Italy) [58]	Non-controlled pilot study (26)	Soratinex^®^ scalp and body cleansing gel; scalp and body ointment and skin conditioner (26)	NA	—PASI ^1^ —Photography analysis —Monitoring ADRs ^3^	6 weeks (baseline; 1, 2, 3, 4, and 6 weeks (total time of observation was 8 weeks))	—After 6 weeks, 3 individuals had no clinical improvements, 6 showed a slight improvement, 9 showed a good response, and 5 achieved an excellent improvement —7 patients reported mild and transient itching and 1 patient with scalp psoriasis discontinued treatment because of folliculitis-like inflammation	—Lack of control group —Small sample size —Short duration of study and follow-up time —Small amount of outcome measures

^1^ PASI: psoriasis area severity index. ^2^ VAS: visual analog scale. ^3^ ADRs: adverse drug reactions. ^4^ PGA: physician global assessment. ^5^ BSA: body surface area. ^6^ DLQI: dermatology life quality index. ^7^ PSI: psoriasis severity index. NA = not applicable.

**Table 6:** Product characteristics of the products used in the included studies focusing on psoriasis. Details about the composition of the preparations are given in Table S1 (Supplementary Materials).

Author(s) (Publication Year; Country)	Intervention (Origin)	Intervention: Self-Made or Purchased	Control	Control: Self-Made or Purchased	Analyses of the EO-containing Product
Thomas et al. (2015; Australia) [56]	20% kānuka oil (from *Kunzea ambigua*)	Prepared in the compounding pharmaceutical laboratory of the School of Pharmacy at the University of Tasmania	The same as the test formulation, but without the kānuka oil component	Prepared in the compounding pharmaceutical laboratory of the School of Pharmacy at the University of Tasmania	Not conducted
Fadaei et al. (2021; Iran) [33]	*Boswellia* spp. (the EO of frankincense and an ethanolic extract, the pulp of *Cucurbita pepo*, the and roots of *Glycyrrhiza glabra*)	Self-made (active products purchased from a local market in Teheran)	Vehicle cream	Self-made	—Organoleptic characteristics —Determination of pH —Mechanical stability —Temperature cycle test —Determination of viscosity —Microbial test —Total phenolic contents
Kohladooz et al. (2018; Iran) [31]	Chamomile-pumpkin (ChP) oleogel (*Matricaria chamomilla* and *Cucurbita pepo* seed)	Self-extracted and self-made (standard pumpkin seed oil was purchased from the Giah Essence Phytopharm Co.)	Vehicle (liquid paraffin)	Self-made	Not conducted
Hercegova et al. (2016; Italy) [57]	Soratinex^®^ scalp and body cleansing gel; scalp and body ointment and skin conditioner	Purchased	NA	NA	Not conducted
Fioranelli et al. (2016; Italy) [58]	Soratinex^®^ scalp and body cleansing gel; scalp and body ointment and skin conditioner	Purchased	NA	NA	Not conducted

NA = not applicable.

**Table 7:** Characteristics, main results, and limitations of the included study focusing on rosacea. Details about the composition of the preparation are given in Table S1 (Supplementary Materials).

Author(s) (Publication Year; Country)	Study Design (n)	Intervention (n)	Control (n)	Outcome Measure(s)	Study Duration (Evaluation Period)	Main Results	Limitations
Ebneyamin et al. (2019; Iran) [59]	Randomized, double-blind, placebo-controlled, prospective clinical trial (35)	2.5% TTO ^1^ in permethrin gel (35)	Vehicle gel (35)	—SSSB ^2^ (mean Dd/cm^2^ ^3^) —Erythema, papule and pustules, flushing, telangiectasia, edema, plaque, and stinging were monitored via photographs —Monitoring ADRs ^4^	12 weeks (baseline; 2, 5, 8, and 12 weeks)	—After 12 weeks, the mean Dd/cm2 ^3^ on the treatment side were significantly decreased compared to the placebo side —After 12 weeks, a significant improvement in the papules and pustules, burning, stinging, and dry appearance were observed on the treatment side and not on the placebo side —Plaques, telangiectasia, flushing, and edema did not change on both sides —Mild-to-moderate ADRs ^4^ were reported by most of the individuals on both sides of their face with skin dryness, erosion, burning, and stinging being prevalent	—Risk of cross-contamination —No demographics reported —Small sample size —No distinction between the effect of permethrin and TTO ^1^

^1^ TTO: tea tree oil. ^2^ SSSB: standard skin surface biopsy. ^3^ Dd/cm^2^: Demodex density per cm^2^. ^4^ ADRs: adverse drug reactions.

**Table 8:** Product characteristics of the product used in the included studies focusing on rosacea. Details about the composition of the preparation are given in Table S1 (Supplementary Materials).

Author(s) (Publication Year; Country)	Intervention (Origin)	Intervention: Self-Made or Purchased	Control	Control: Self-Made or Purchased	Analyses of the EO-Containing Product
Ebneyamin et al. (2019; Iran) [59]	2.5% permethrin with TTO ^1^ gel (*Melaleuca alternifolia*)	Not mentioned	Vehicle gel	Not mentioned	Not conducted

^1^ TTO: tea tree oil.

## Data Availability

Requests to access the datasets may be directed to the first author.

## Supplementary Materials

The following supporting information can be downloaded at https://www.mdpi.com/article/10.3390/ph17050571/s1. Table S1: composition of the preparations as described in the corresponding references; and Table S2: search strategies used for each electronic database.
