# Supplementary material for: The Therapeutic Potential of Essential Oils in Managing Inflammatory Skin Conditions: A Scoping Review
Source: Pharmaceuticals (Basel). 2024 Apr 29;17(5):571. doi: 10.3390/ph17050571 (PMC11123799; doi:10.3390/ph17050571)
Supplement: Supplementary file 1 [file pharmaceuticals-17-00571-s001.zip › pharmaceuticals-2973600-supplementary.pdf]

Scoping Review – Supplementary Materials

The Therapeutic Potential of Essential Oils in Managing Inflammatory Skin Conditions: a Scoping Review

Anouk E.W.K. Dontje [1], Catharina C. M. Schuiling-Veninga [2], Florence P.A.M. van Hunsel[2,3], Corine Ekhart[3], Fatih Demirci[4], Herman J. Woerdenbag[1*]

[1] Department of Pharmaceutical Technology and Biopharmacy, Groningen Research Institute of Pharmacy (GRIP), University of Groningen, Antonius Deusinglaan 1, 9713 AV Groningen, The Netherlands; anoukdontje@gmail.com (A.E.W.K.D.)
[2] Department of Pharmacotherapy, -Epidemiology, and -Economy, Groningen Research Institute of Pharmacy (GRIP), University of Groningen, Antonius Deusinglaan 1, 9713 AV Groningen, The Netherlands; c.c.m.schuiling-veninga@rug.nl (C.C.M.S.-V.)
[3] Netherlands Pharmacovigilance Centre Lareb, Goudsbloemvalei 7, 5237 MH 's-Hertogenbosch, The Netherlands; f.vanhunsel@lareb.nl (F.P.A.M.v.H.); c.ekhart@lareb.nl (C.E.)
[4] Department of Pharmacognosy, Faculty of Pharmacy, Anadolu University, 26470 Eskisehir, Türkiye; demircif@gmail.com (F.D.)
* Correspondence: h.j.woerdenbag@rug.nl (H.J.W.); Tel.: +31-6-31921365

Abstract: Conventional therapy is commonly used for the treatment of inflammatory skin conditions, but undesirable effects, such as erythema, dryness, skin thinning, and resistance to treatment, may cause poor patient compliance. Therefore, patients may seek for complementary treatment with herbal plant products including essential oils (EOs). This scoping review aims to generate a broad overview of EOs used to treat inflammatory skin conditions, being acne vulgaris, dermatitis and eczema, psoriasis, and rosacea, in a clinical setting. The quality, efficacy, and safety of various EOs and preparations thereof are reviewed, and the potential as well as limitations of EOs in the treatment of inflammatory skin conditions are discussed. Twenty-nine eligible studies (case studies, uncontrolled clinical studies, randomized clinical studies) on EO applications for inflammatory skin conditions were retrieved from scientific electronic databases (PubMed, Embase, Scopus, Cochrane Library). As an initial result, tea tree (*Melaleuca alternifolia*) oil emerged as the most studied EO. Clinical studies with tea tree oil gel for acne treatment showed efficacy with fewer adverse reactions compared to conventional treatment. Uncontrolled studies indicated the potential efficacy of ajwain (*Trachyspermum ammi*) oil, eucalyptus (*Eucalyptus globulus*) oil, and cedarwood (*Cedrus libani*) oil in the treatment of acne, but further research is required to reach conclusive evidence. Placebo-controlled studies revealed positive effects of kānuka (*Kunzea ericoides*) oil and frankincense (*Boswellia* spp.) oil in the treatment of psoriasis and eczema. The quality verification of the EO products was inconsistent, with some studies lacking analyses and transparency. Quality limitations of some studies included a small sample size, a short duration, and the absence of a control group. The present review underscores the need for extended, well-designed clinical studies to further assess EO efficacy and safety for treating inflammatory skin conditions with products of ensured quality, and to further elucidate the mechanisms of action involved.

Keywords: acne vulgaris; case reports; clinical studies; dermatitis; eczema; efficacy; essential oils (EOs); psoriasis; rosacea; quality; tea tree oil (TTO)

Table of Contents:

Table S1: Composition of the preparations as described in the corresponding references.
Table S2: Search strategies used per electronic database**.**

Table S1: Composition of the preparations as described in the corresponding references.

Note that there are discrepancies in the information provided in the various articles. Not always Latin binomials but trivial names (in English) are used for the plants; if Latin binomial names are used, an author name behind the species name often lacks; the base (type, composition) used in a formulation is not always clearly described.

Author(s) (Publication year; Country)	Product name	Constituents	Amount
Kwon *et al.* (2014; South Korea) [37]	*Lactobacillus*-fermented *Chamaecyparis obtusa* (LFCO) 5% in cream	Water	63.4%
		Chamaecyparis obtusa ferment filtrate	5.0%
		Cyclopentasiloxane	4.0%
		Alcohol	3.5%
		Butylene glycol	3.0%
		Dimethicone	3.0%
		Cyclopentasiloxane	3.0%
		Polysilicone-11	3.0%
		Sodium hydroxide	3.0%
		Cetyl PEG/PPG-10/1 Dimethicone	2.0%
		Dimethicone/Vinyl dimethicone crosspolymer	2.0%
		Glycerin	1.2%
		Polysorbate 60	1.0%
		Sodium C8-16 isoalkylsuccinyl lactoglobulin sulfonate	1.0%
		Sodium hyaluronate	1.0%
		Xanthan gum	0.8%
	Tea tree oil 5% in cream	Water	63.4%
		Melaleuca alternifolia essential oil	5.0%
		Cyclopentasiloxane	4.0%
		Alcohol	3.5%
		Butylene glycol	3.0%
		Dimethicone	3.0%
		Cyclopentasiloxane	3.0%
		Polysilicone-11	3.0%
		Sodium hydroxide	3.0%
		Cetyl PEG/PPG-10/1 dimethicone	2.0%
		Dimethicone/Vinyl dimethicone crosspolymer	2.0%
		Glycerin	1.2%
		Polysorbate 60	1.0%
		Sodium C8-16 isoalkylsuccinyl lactoglobulin sulfonate	1.0%
		Sodium hyaluronate	1.0%
		Xanthan gum	0.8%
Da Silva *et al.* (2012; Brazil) [38]	1% Copaiba essential oil in natrozol gel	*Copaifera langsdorffii* essential oil	1.0%
		Tween 80	0.5%
		Parabens	
		Sorbic acid	
	Natrozol gel	Tween 80	0.5%
		Parabens	
		Sorbic acid	
Ghovatti *et al.* (2019; Iran) [40]	Cinnamon gel 0.5%	*Cinnamon verum* bark essential oil	0.5 g
		Water	3.8 g
		Propylene glycol	0.02 g
		Methylparaben	0.18 g
		EDTA	0.75 g
		Carbomer 940	1.0 g
		Propylene glycol	10 g
		Ethanol	64.37 g
Talebi *et al.* (2020; Iran) [41]	1% (w/w) Ajwain gel	*Trachyspermum ammi* essential oil	1.4%
		Propylene glycol	50 g
		96% Ethanol	320.8 g
		Distilled water	199 g

		Carbomer 941<br>Triethanolamine	5 g
Hassan *et al.* (2013; Saudi Arabia) [43]	Eucalyptus oil cream	Emulsifying wax<br>White soft paraffin<br>Liquid paraffin<br>Eucalyptus oil<br>Purified water	10 g<br>50 g<br>20 g<br>2 g<br>To 100 g
Najafi-Taher *et al.* (2022; Iran) [44]	Tea tree oil nano-emulsion containing adapalene gel	*Melaleuca alternifolia* essential oil<br>Adapalene<br>Dimethylsulfoxide<br>Ethanol<br>Tween20:Span80	6%<br>0.1%<br>10%<br>10%<br>20%
Mazzarello et al. (2018; Italy) [46]	PATC cream	Propolis extract<br>*Melaleuca alternifolia* essential oil<br>*Aloe vera* leaf juice<br>Vehicle cream	20%<br>3%<br>10%
	Erythromycin cream	Erythromycin<br>Vehicle cream	3%
Mazzarello *et al.* (2020; Italy) [47]	MOTC cream	*Myrtus communis* essential oil<br>*Origanum vulgare* essential oil<br>Tretinoin<br>Vehicle cream	3.74%<br>0.1%<br>0.025%
	CTG cream	Clindamycin<br>Tretinoin<br>Vehicle cream	1%<br>0.025%
Parveen *et al.* (2009; India) [48]	Cream base	Stearic acid<br>Potassium hydroxide<br>Cetostearyl alcohol<br>Bees wax (white)<br>Triethanolamine<br>Glycerin<br>Methylparaben sodium<br>Propylparaben sodium<br>Liquid paraffin<br>Lanolin<br>Petroleum jelly<br>Purified water	20 g<br>3 g<br>7 g<br>5 g<br>1.5 g<br>5 g<br>200 mg<br>200 mg<br>6 g<br>2 g<br>4 g<br>100 ml
	Unani herbomineral cream	*Iris ensata* Thunb.<br>*Azadirachta indica* A. Luss<br>*Cyperus rotundus* Linn.<br>*Nuctanthes arbortristis* Linn.<br>*Astragalus sarcocolla* Dymock<br>*Aloe barbadensis* Mill.<br>*Matricaria chamomilla* Linn.<br>*Vetiveria zizanoides* (Linn.) Nash<br>*Commiphora myrrha* (Nees) Engl.)<br>*Bosellia serrata* Roxb.<br>*Jasminum officinalis* Linn.<br>*Prunus dulcis* var. *dulcis* (D.C.) Buchheim<br>*Olea europaea* Linn<br>Zinc oxide<br>Cream base	1.5%<br>1.5%<br>1.5%<br>1.5%<br>1.5%<br>3.0%<br>0.5%<br>0.3%<br>0.2%<br>0.2%<br>0.3%<br>5.0%<br>5.0%<br>1.5%<br>q.s.
Behehsti Roy *et al.* (2014; Iran) [53]	5% Tea tree oil gel	*Melaleuca alternifolia* essential oil<br><br>Terpinen-4-ol<br>γ-Terpinene<br>α-Terpinene<br>1.8-Cineole<br>Terpinolene<br>p-Cymene<br>α-Pinene<br>α-Terpineol<br>Aromadendrene<br>δ-Cadinene	 <br>41.1%<br>21.0%<br>11.4%<br>4.7%<br>2.4%<br>2.6%<br>1.9%<br>3.1%<br>1.7%<br>1.0%

		Limonene<br>Vehicle gel	0.9%
	Vehicle gel	Hydroxypropyl cellulose	
De Valois (2004; United Kingdom) [54]	Base shampoo + essential oil formulation and a scalp soak	Geranium<br>Palmarosa<br>Tea tree<br>Spike lavender<br>German chamomille<br>Peppermint	20%<br>25%<br>20%<br>20%<br>10%<br>5%
Allan (2003; Unknown) [55]	Base shampoo + drops of essential oil mixture & 2% dilution of the essential oil mixture in an aqueous based cream	Geranium<br>Palmarosa<br>Laurel<br>Tansy<br>Spike lavender	
Thomas *et al.* (2015; Australia) [56]	20% Kānuka oil (ointment and/or scalp lotion)	Kānuka oil<br>Vehicle	20%
	The same as the test formulation, but without the kānuka oil component (ointment and/or scalp lotion)	Salicylic acid<br>Liquor carbonis detergens<br>Cod liver oil<br>Vanillin	3%
Fadaei *et al.* (2021; Iran) [33]	Boswellia-based cream	*Cucurbita pepo* oil<br>*Boswellia* spp. ethanolic extract<br>*Boswellia* spp. oil<br>*Glycyrrhiza glabra* aqueous extract<br>Vehicle	10.0%<br>5.0%<br>10.0%<br>0.2%<br>74.8%
	Vehicle cream	Tween 80<br>Span60<br>BHT<br>Glycerin<br>Ethanol<br>Methylparaben<br>Propylparaben<br>Water	4.5%<br>2.5%<br>0.2%<br>10.0%<br>10.0%<br>0.18%<br>0.02%<br>Up to 100%
Kohladooz *et al.* (2018; Iran) [31]	ChP oleogel	*Matricaria chamomilla* oil<br>*Cucurbita pepo seed* oil<br>Colloidal silicon dioxide	47.5%<br>47.5%<br>5.0%
	Vehicle gel	*Matricaria chamomilla* oil<br>*Cucurbita pepo seed* oil<br>Silicon dioxide<br>Liquid paraffin	0.5%<br>0.5%<br>5.0%<br>94%
Hercegova *et al.* (2016; Italy) [57] & Fioranelli *et al.* (2016; Italy) [58]	Soratinex® scalp and body cleansing gel	Salicylic acid<br>Citric acid<br>Glycolic acid	
	Soratinex® scalp and body ointment	Paraffinum liquid<br>Paraffinum solidum<br>Solanum tuberosum<br>Zinc oxide<br>Salicylic acid<br>*Prunus amygdalus dulcis* oil<br>*Simmondsia chinensis* oil<br>*Persea gratissima* oil<br>*Daucus carota* oil<br>*Calendula officinalis* extract<br>*Citrus sinensis* oil<br>*Triticum vulgare* germ oil	

		Prunus armeniaca kernel oil<br>*Lavendula* augustifolia<br>*Santalum* album oil<br>*Pogostemon* cablin oil<br>*Pelargonium* graveolens<br>*Rosemary officinalis* extract<br>*Dromiceius* oil<br>*Citrus aurantium* ssp bergamia oil<br>*Pinus sylvestris* leaf oil<br>*Chamomilla recutita* oil<br>*Commiphora myrrha* oil<br>*Citrus aurantium amara* flower oil	
	Soratinex® skin conditioner	Olive oil<br>Sesame seed oil<br>Emu oil<br>Lavender oil<br>Eucalyptus oil<br>Natural vitamin E	
Ebneyamin *et al.* (2019; Iran) [59]	Permethrin 2.5% with Tea tree oil gel	Tea tree oil<br>Permethrin (99.88% purified)<br>Vehicle	
	Vehicle gel	Carbomer 941<br>Benzalkonium chloride<br>Triethanolamine	
Orafidiya *et al.* (2002; Nigeria) [50]	*Ocimum gratissimum* L. leaf essential oil test preparations	0.5% Ocimum oil (African basil) in 1% v/v polysorbate 80<br>1% Ocimum oil in 1% v/v polysorbate 80<br>2% Ocimum oil in 1% v/v polysorbate 80<br>5% Ocimum oil in 1% v/v polysorbate 80<br>0.5% Ocimum oil in cetomacrogol blend<br>1% Ocimum oil in cetomacrogol blend<br>2% Ocimum oil in cetomacrogol blend<br>5% Ocimum oil in cetomacrogol blend<br>0.5% Ocimum oil in petrolatum base<br>1% Ocimum oil in petrolatum base<br>2% Ocimim oil in petrolatum base<br>5% Ocimum oil in petrolatum base<br>0.5% Ocimum oil in 50% v/v alcohol (= ethanol)<br>1% Ocimum oil in 50% v/v alcohol<br>2% Ocimum oil in 50% v/v alcohol<br>5% Ocimum oil in 50% v/v alcohol	
Orafidiya *et al.* (2004; Nigeria) [51]	*Ocimum gratissimum* L. leaf essential oil test preparations	40% Arachis oil in 1% polysorbate 80 (placebo)<br>Neat aloe gel dispersed in 1% polysorbate 80 (negative control)<br>1% Clindamycin (reference drug product; positive control)<br>2% Ocimum oil lotion<br>2% Ocimum oil lotion in 25% aqueous aloe gel<br>2% Ocimum oil lotion in 50% aqueous aloe gel<br>2% Ocimum oil lotion in 100% aloe gel	

Table S2: Search strategies used per electronic database**.**

Electronic Database	Search Strategy
PubMed	1. ("Oils. Volatile"[MESH] OR volatile oils[tiab] OR volatile oil[tiab] OR oil. essential[tiab] OR oils. essential[tiab] OR essential oils[tiab] OR oils. volatile[tiab])<br>2. ("Plant oils"[MESH] OR oils. plant[tiab] OR plant oil[tiab] OR plant oils[tiab] OR oil. plant[tiab] OR oils. vegetable[tiab] OR vegtable oil[tiab] OR vegtable oils[tiab] OR oil. vegetable[tiab])<br>3. ("Acne Vulgaris" [MESH] OR acne[tiab])<br>4. ("Psoriasis" [MESH] OR psoriases[tiab] OR pustulosis of palms and soles[tiab] OR pustulosis palmaris et plantaris[tiab] OR palmoplantaris pustulosis[tiab] OR pustular psoriasis of palms and soles[tiab])<br>5. ("Dermatitis" [MESH] OR dermatitides[tiab])<br>6. ("Rosacea" [MESH] OR acne rosacea[tiab] OR phymatous rosacea[tiab] OR rosacea. phymatous[tiab] OR papulopustular rosacea[tiab] OR rosacea. papulopustular[tiab] OR erythematotelangiectatic rosacea[tiab] OR rosacea. erythematotelangiectatic[tiab] OR granulomatous rosacea[tiab] OR rosacea. granulomatous[tiab])<br>7. (#1 OR #2) AND (#3 OR #4 OR #5 OR #6)
Embase	1. ("Oils. Volatile"[MESH] OR volatile oils[tiab] OR volatile oil[tiab] OR oil. essential[tiab] OR oils. essential[tiab] OR essential oils[tiab] OR oils. volatile[tiab])<br>2. ("Plant oils"[MESH] OR oils. plant[tiab] OR plant oil[tiab] OR plant oils[tiab] OR oil. plant[tiab] OR oils. vegetable[tiab] OR vegtable oil[tiab] OR vegtable oils[tiab] OR oil. vegetable[tiab])<br>3. ("Acne Vulgaris" [MESH] OR acne[tiab])<br>4. ("Psoriasis" [MESH] OR psoriases[tiab] OR pustulosis of palms and soles[tiab] OR pustulosis palmaris et plantaris[tiab] OR palmoplantaris pustulosis[tiab] OR pustular psoriasis of palms and soles[tiab])<br>5. ("Dermatitis" [MESH] OR dermatitides[tiab])<br>6. ("Rosacea" [MESH] OR acne rosacea[tiab] OR phymatous rosacea[tiab] OR rosacea. phymatous[tiab] OR papulopustular rosacea[tiab] OR rosacea. papulopustular[tiab] OR erythematotelangiectatic rosacea[tiab] OR rosacea. erythematotelangiectatic[tiab] OR granulomatous rosacea[tiab] OR rosacea. granulomatous[tiab])<br>7. (#1 OR #2) AND (#3 OR #4 OR #5 OR #6)
Cochrane Library	1. ('oils. volatile' OR 'volatile oils' OR 'volatile oil' OR 'oil. essential' OR 'oils. essential' OR 'essential oils')<br>2. ('oils. plant' OR 'plant oil' OR 'plant oils' OR 'oil. plant' OR 'oils. vegetable' OR 'vegtable oil' OR 'vegtable oils' OR 'oil. vegetable')<br>3. ('acne vulgaris' OR acne)<br>4. (psoriasis OR psoriases OR 'pustulosis of palms and soles' OR 'pustulosis palmaris et plantaris' OR 'palmoplantaris pustulosis' OR 'pustular psoriasis of palms and soles')<br>5. ("Dermatitis" OR dermatitides)<br>6. ("Rosacea" OR acne rosacea OR phymatous rosacea OR rosacea. phymatous OR papulopustular rosacea OR rosacea. papulopustular OR erythematotelangiectatic rosacea OR rosacea. erythematotelangiectatic OR granulomatous rosacea OR rosacea. granulomatous)<br>7. (#1 OR #2) AND (#3 OR #4 OR #5 OR #6)

Scopus	1. (“oils. volatile” OR “volatile oils” OR “volatile oil” OR “oil. essential” OR “oils. essential” OR “essential oils”)<br>2. (“oils. plant” OR “plant oil” OR “plant oils” OR “oil. plant” OR “oils. vegetable” OR “vegtable oil” OR “vegtable oils” OR “oil. vegetable”)<br>3. (“acne vulgaris” OR acne)<br>4. (psoriasis OR psoriases OR “pustulosis of palms and soles” OR “pustulosis palmaris et plantaris” OR “palmoplantaris pustulosis” OR “pustular psoriasis of palms and soles”)<br>5. (“Dermatitis” OR dermatitides)<br>6. (“Rosacea” OR acne rosacea OR phymatous rosacea OR rosacea. phymatous OR papulopustular rosacea OR rosacea. papulopustular OR erythematotelangiectatic rosacea OR rosacea. erythematotelangiectatic OR granulomatous rosacea OR rosacea. granulomatous)<br>7. (#1 OR #2) AND (#3 OR #4 OR #5 OR #6)
